# A Potential Mechanism of Kidney-Tonifying Herbs Treating Unexplained Recurrent Spontaneous Abortion: Clinical Evidence From the Homogeneity of Embryo Implantation and Tumor Invasion

**DOI:** 10.3389/fphar.2021.775245

**Published:** 2022-01-26

**Authors:** Hang Zhou, Yi Yang, Linwen Deng, Yongqing Yao, Xin Liao

**Affiliations:** ^1^ School of Basic Medical Sciences, Chengdu University of Traditional Chinese Medicine, Chengdu, China; ^2^ Department of Gynecology, Hospital of Chengdu University of Traditional Chinese Medicine, Chengdu, China; ^3^ Department of Information, Hospital of Chengdu University of Traditional Chinese Medicine, Chengdu, China

**Keywords:** unexplained recurrent spontaneous abortion, tumor invasion, clinical trials and validation experiments, kidney-tonifying herbs, embryo implantation

## Abstract

**Background:** Kidney-tonifying herbs (KTHs) are widely used to treat unexplained recurrent spontaneous abortion (URSA) based on the theory of traditional Chinese medicine (TCM). However, there is still a lack of systematic evaluation and mechanistic explanation for these treatments.

**Objective:** The purpose of this study was to assess the clinical efficacy, and to investigate the potential mechanisms, of KTH based on TCM for the treatment of URSA.

**Methods:** A systematic literature search was conducted within PubMed, Embase, China Biomedical Literature database, Web of Science (WOS), China National Knowledge Infrastructure (CNKI) database, and the Wanfang database to find articles reporting on the Chinese herbal formula based around KTH for treating URSA, which were published between January 2010 and June 2021. A full bibliometric analysis was carried out; in addition, randomized controlled trial (RCT) articles were selected for systematic evaluation and meta-analysis. The drugs with the highest frequency of KTHs were screened for meta-analysis. Finally, network analysis and molecular docking were used to study the key components and potential pathway of KTHs in the treatment of URSA.

**Results:** The meta-analysis included nine RCTs involving 1,054 subjects. Compared with the control groups, the clinical efficacy of TCM-based KTHs in the treatment of URSA patients significantly improved outcomes. Additionally, a component target pathway network was identified, which included 32 potential blood activating components and 113 main targets. Japonine, sopranol, lysine, and matrine were considered the most important bioactive molecules for KTHs. The key potential therapeutic pathway for URSA was a tumor-related signaling pathway. The target genes for URSA regulated by KTHs were highly similar to tumor biological processes such as the regulation of apoptotic signaling pathways, inflammatory responses, angiogenesis, and epithelial metabolic transition.

**Conclusion:** KTH has great potential for treating URSA. Because the maintenance of pregnancy has a high similarity with tumor invasion, the research relating to tumor mechanisms should also be followed up as it may lead to new ideas and breakthroughs for research into URSA. At the same time, embryonic and decidual cells share a high degree of cellular heterogeneity and spatial structural complexity with tumor cells, and a single cell combined with spatial omics may be the best future approach for validating KTH mechanisms.

## Introduction

Recurrent spontaneous abortion (RSA) is challenging to diagnose and treat. Its classic definition is two or more clinical continuous abortions before 20 weeks of pregnancy in fertile couples (Practice Committee of the American Society for Reproductive Medicine, 2012; Colley et al., 2019). The etiology of RSA includes genetic abnormalities, endocrine disorders, anatomical abnormalities, infectious, prothrombotic state, and immune factors (Li et al., 2002; Qian et al., 2018). Its incidence rate ranges approximately from 1% to 5% in women during their childbearing years (Nigro et al., 2011; Ewington et al., 2019; Homer 2019). However, about 50% of RSAs are still undiagnosed and/or untreated. This condition is often referred to as unexplained recurrent spontaneous abortion (URSA) after exclusion of diagnosis and is considered an early spontaneous abortion within the first 12 weeks of pregnancy (Pereza et al., 2017; ESHRE Guideline Group on RPL et al., 2018). The disease has serious physical and mental impacts on the patients and their families (Cao et al., 2017; Tavoli et al., 2018). Therefore, it is necessary to study effective treatment methods to reduce pregnancy loss and to help maintain pregnancy in URSA patients (Zhang et al., 2021). The current therapeutic options for URSA mainly include preimplantation genetic screening (PGS), suppression of alloimmunity, and anticoagulant therapy (Rey et al., 2003; Qin et al., 2016; Zhao et al., 2017; Ding et al., 2019; Feng, 2019; Qin et al., 2020; Zhang et al., 2021). Cyclosporine A (Zhou et al., 2007), intravenous immunoglobulin (Wang et al., 2016; Muyayalo et al., 2018), lymphocyte active immunity, and glucocorticoids are the main regimens used to suppress alloimmunity, but the effectiveness and safety of these regimens have not been fully validated using large sample clinical studies. Therefore, until now, there has been a lack of unified diagnostic criteria and efficient treatments for URSA (Mekinian et al., 2016; Li et al., 2020).

In recent years, traditional Chinese medicine (TCM) has been accepted as a mainstream of medical care, and has become a popular supplement to Western medicine for the treatment of URSA (Fujii et al., 2000; Li et al., 2014; Yang et al., 2018). Kidney-tonifying herbs (KTHs) are the most commonly used prescription for TCM-based treatment (Li et al., 2012; Li et al., 2016). In the past few years, the data accumulated from personal clinical experience, case reports, noncontrolled trials, animal experiments, and randomized controlled trials (RCT) show that when treating URSA, KTHs alone and KTHs combined with Western medicine have similar effects regarding the improvement of pregnancy outcomes and symptoms (Liu et al., 2009; Zhao et al., 2018; Feng 2019; Zhang et al., 2019). Although KTHs are widely used in patients with URSA, it is difficult for KTHs to be recognized internationally due to the complexity of its ingredients and a lack of pharmacological mechanisms.

**FIGURE 1:**
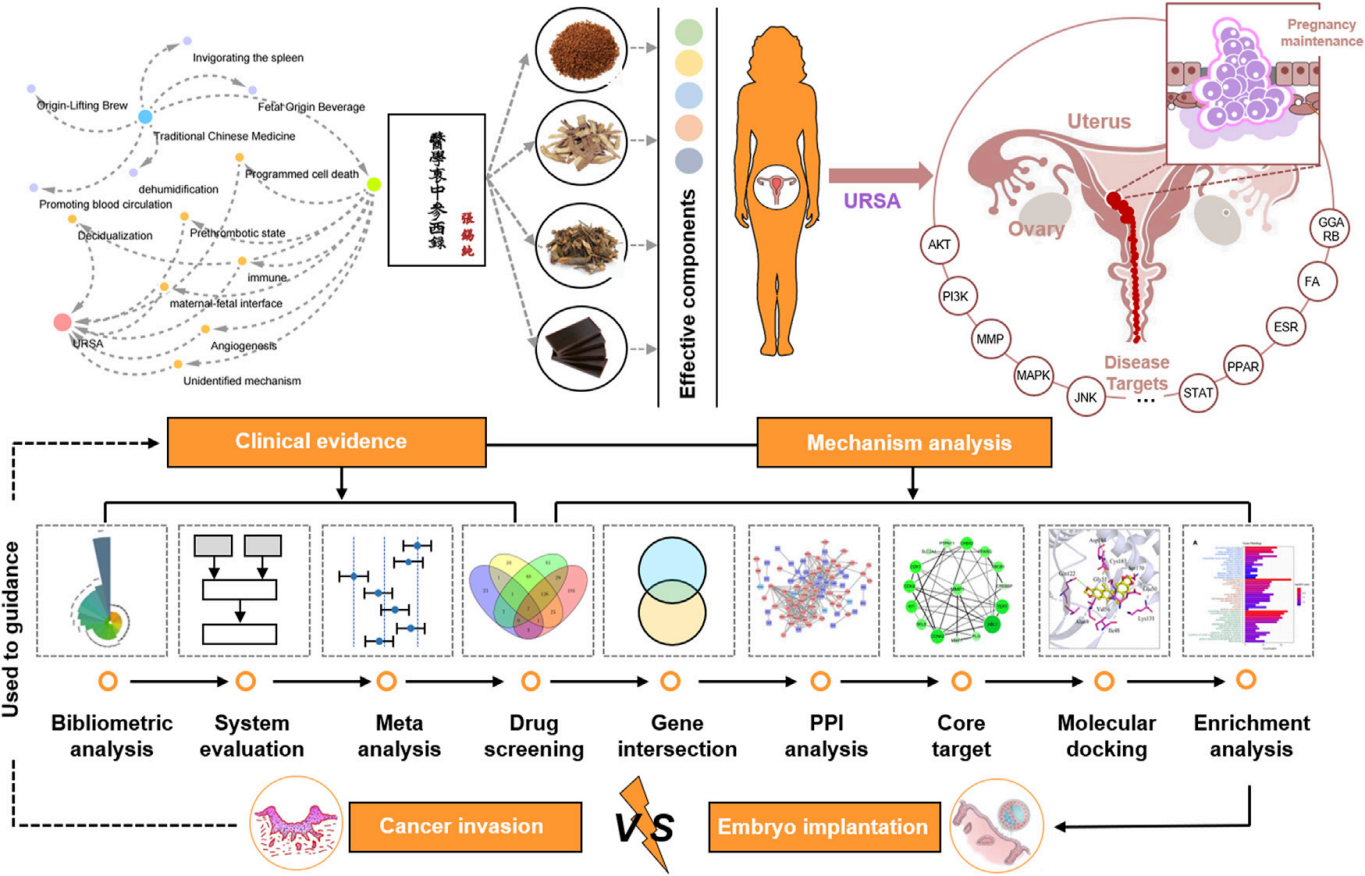
Clinical evidence and underlying mechanisms of kidney-tonifying herbs (KTHs) for treating unexplained recurrent spontaneous abortion (USRA).

At present, it is known that embryo implantation is highly similar to cancer invasion. The phenotypes of apoptosis, inflammation, proliferation, invasion, adhesion, and angiogenesis in the interactions between embryonic trophoblast cells and endometrial epithelial cells at the maternal–fetal interface are highly similar to cancer processes (Murray and Lessey, 1999; Hannan and Salamonsen, 2008; Perry et al., 2009; Zhang et al., 2020). According to previous studies, we hypothesized that the mechanism of TCM for tocolysis may be similar to that observed in tumor activation. A key difference between them lies in the gene targets and biological process affected by the medicine. The goal of this study was to test this hypothesis. Specifically, bibliometric analysis, meta-analysis, network pharmacology, and molecular docking were conducted to examine the efficacy of the published TCM-based KTHs preparations and the control groups for treating URSA. The research also identified the potential pathways of TCM kidney-tonifying prescriptions in the treatment of URSA, and provided evidence-based scientific support for URSA treatment in clinical practice (Figure 1).

## Data and methods

### Bliometric Analysis of Unexplained Recurrent Spontaneous Abortion Treated by Traditional Chinese Medicine in the Last 10 Years

We conducted a systematic literature search in the following clinical research databases: PubMed, Embase, Cochrane Library, Web of Science (WOS), China National Knowledge Infrastructure (CNKI), Wanfang Database, China Biomedical Database (CBM), and the China Science Journal Database (VIP), from the establishment of each database through June, 2021. We developed a search formula for the treatment of URSA by KTHs based on the PICOS strategy (Detailed strategies are provided in Supplementary Data Sheet S1), and then collated the screened literature using ENDNOTE software.

We also established a bibliometric analysis strategy for the CNKI and WOS databases (Supplementary Data Sheet S2). Between (“2011-01-01,” “2021-06-01”) and (literature classification was limited to traditional Chinese medical science, Chinese herb and integrated traditional Chinese and Western medicine); Search scope: general database. After downloading the refworks format, the synonyms were converted into Chinese and English, and the synonyms were clustered uniformly, including KTHs. The drug was defined as “KTH”; the selection used a modified g-index in each slice:

$$g^{2}\leq k\sum i_{\leq g}c_{i}k\in Z^{+}.$$

To include more or fewer nodes, increase or decrease the scale factor k = 25. The keywords co-occurrence analysis, cluster analysis, mutation term analysis, as well as time line graph analysis were performed using CiteSpace (Version 5.8.R1).

### Clinical Evidence of Kidney-Tonifying Herbs in the Treatment of Unexplained Recurrent Spontaneous Abortion

The systematic review and meta-analysis were conducted in accordance with the PRISMA guidelines (Moher et al., 2009; McInnes et al., 2017). The detailed research process is illustrated in the Supplementary Materials. All randomized controlled trials (RCTs) were included to study the efficacy of KTHs alone or combined with modern drugs, in the treatment of URSA. Studies reported in languages besides Chinese and English were excluded. Nonrandomized controlled trials or animal experiments were excluded.

### Participant Inclusion

Patients included were in their first 3 months of pregnancy, had a pregnancy confirmed by serum human chorionic gonadotropin (hCG) or ultrasound, and had a history of URSA diagnosis, which was defined as two or more spontaneous abortions. The following four causes were excluded: infection, abnormal parental karyotype, endocrine imbalance, and anatomical abnormalities, regardless of the maternal or gestational age, race or nationality, educational level, or economic status. The inclusion criteria also ensured that no treatment was received before pregnancy or before entering the trial. Trials with recurrent spontaneous abortion and nonpregnant URSA participants with a definite etiology were also excluded.

### Types of Intervention

The treatment of interest was KTHs, regardless of dose, administration methods, administration time, or whether KTHs were used in combination with Western medicine. The KTH group was compared with a treatment using only Western medicine. A randomized controlled trial was excluded if the Western medicine changed within the control group. Studies using only bed rest and/or psychological support were also excluded.

### Measurement of Treatment Outcomes

The primary outcome was the clinical response rate and pregnancy outcome reported in the trials. The secondary outcomes included reported hormone levels, serum immunological parameters, and incidence of adverse events during treatment.

### Search Strategy

The search strategy was the same as the bibliometric analysis. The reference list of all identified articles was also manually searched to find possible related studies to supplement the relevant literature.

### Data Extraction and Quality Assessment

Two reviewers (Hang Zhou and Yi Yang) extracted the general information from eligible studies through a predesigned standardized data extraction table: first author, year of publication, TCM syndrome difference, sample size, age, abortion frequency, definition of abortion and live birth, intervention time, treatment intervention and control group, treatment time, and results. Any inconsistencies were resolved by a third reviewer (Yongqing Yao). The methodological quality of each individual study was independently evaluated by two researchers (Yi Yi and Hang Zhou) referring to the Cochrane Handbook (Cumpston et al., 2019; Propadalo et al., 2019) for systematic review of interventions. We used the following criteria for evaluation: random sequence generation, allocation concealment, blinding of participants and personnel, blinding of outcome evaluation, incomplete outcome data, selective reporting, and other biases. Each study was classified as either low-risk, high-risk, or unclear. If there were differences in opinion, the third researcher was referred to (Yongqing Yao).

### Statistical Analysis

A meta-analysis was conducted using the Review Manager (Revman) (computer program; version 5.3, Copenhagen: Nordic Cochrane Center, Cochrane Collaboration, 2014). The relative risk (RR) of 95% confidence interval (CI) was used for binary variables, while weighted mean variance (WMD) and 95% confidence intervals were used for continuous variables. Cochrane’s *p*-value and I2 were used to test the heterogeneity of the study.

### Network Pharmacological Mechanism of Kidney-Tonifying Herbs in the Prevention and Treatment of Unexplained Recurrent Spontaneous Abortion

#### Drug Composition and Target Screening

Drug screening was carried out for the selected literature, and the results were recorded in the tcmsp database (http://tcmspw.com/tcmsp.php) and the Batman database (http://bionet.ncpsb.org/batman-tcm/). The structures of the above components were obtained from the PubChem database and imported into the Swiss target prediction database (http://www.swisstargetprediction.ch/). The targets with prediction scores greater than 0 were selected as drug targets. OMIM (https://omim.org/) and the Genecards database (https://www.genecards.org/) were also searched with the keywords “unexplained recurrent spontaneous abortion” and the disease targets were obtained. The drug targets and disease targets were integrated, and then gene intersection was performed.

#### PPI Network Construction and Core Target Analysis

We searched the above drug disease common targets using the string database (https://string-db.org) (Von Mering et al., 2003). The protein type was set as “*Homo sapiens*.” The minimum interaction threshold was set at 0.4. After the construction of the PPI network of protein interaction, Mcode module was used to analyze gene clusters and screen core targets.

#### Molecular Docking

According to the CAS number of small molecules, the 3D structure of small molecules in SDF format was downloaded from the PubChem database, imported into chembio3d ultra 14.0 for energy minimization, and autodock tools-1.5.6 for hydrogenation, charge calculation, charge distribution, and rotatable key setting. The key target proteins were downloaded from the database PDB (http://www.rcsb.org/), and the crystal water and original ligands were removed using pymol2.3.0. They were then hydrogenated, the charge was calculated and distributed, and the atomic type was specified. AutoDock Vina1.1.2 was used for molecular docking, and PyMOL2.3.0 was used to analyze the interaction mode of the docking results.

#### Enrichment Analysis of GO and KEGG

In metascape and R software, the Bioconductor bioinformatics software package was used to analyze the function enrichment of key target genes GO and KEGG with a *p*-value <0.05 and a *Q*-value <0.05, the results were output in the form of bar and bubble charts. A heatmap was plotted using http://www.bioinformatics.com.cn, an online platform for data analysis and visualization. According to the results of the enrichment analysis, the network diagram for traditional Chinese medicine–components–targets–pathways–phenotypes–diseases was constructed using Cytoscape.

## Results

### Results of Bibliometric Analysis

**FIGURE 2:**
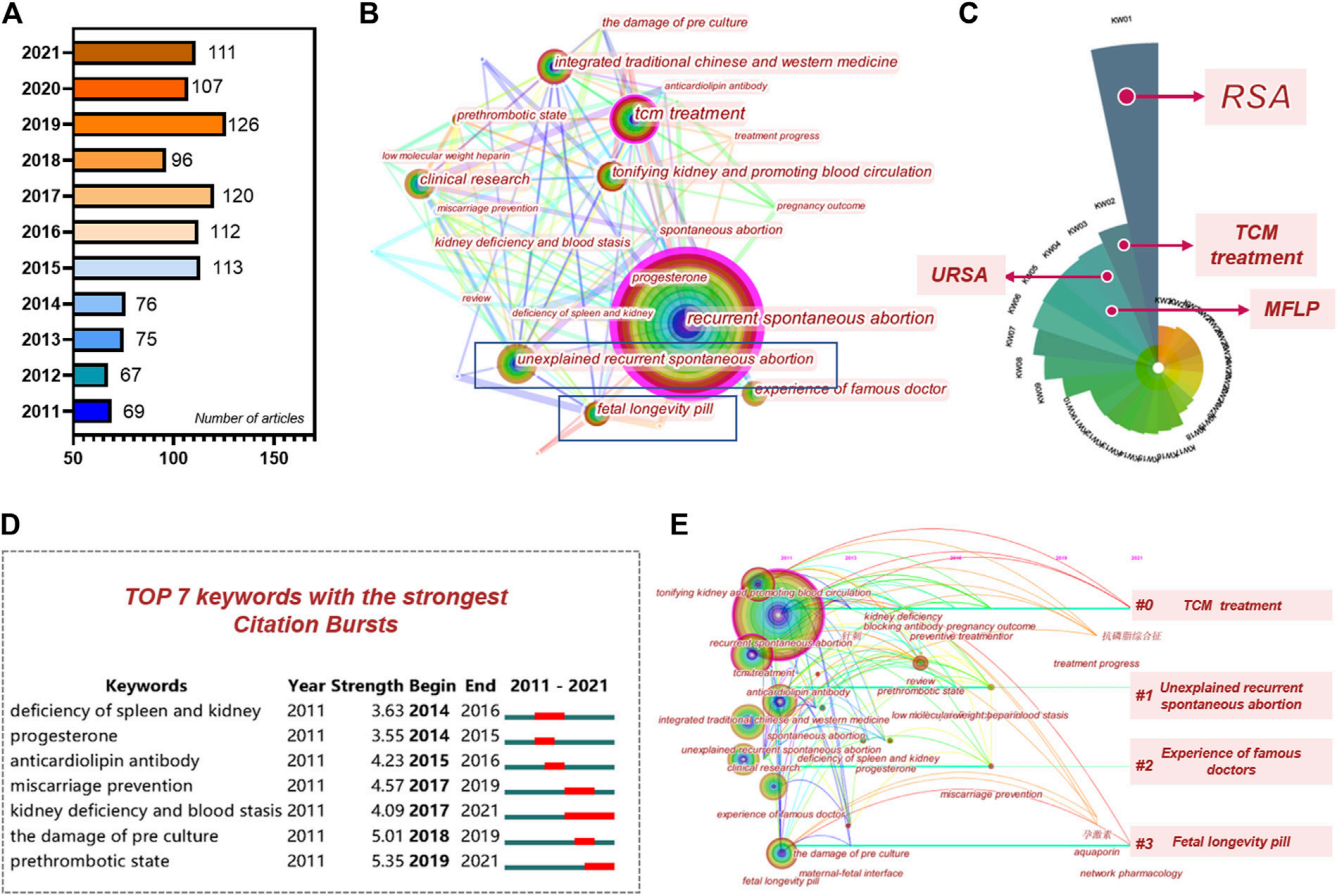
Bibliometric analysis of KTHs on abortion. **(A)** Number of articles published in time. **(B)** Keyword colinear analysis. **(C)** Polar coordinate histogram. **(D)** Keyword co-occurrence chart. **(E)** Keyword clustering analysis timeline chart.

A total of 1,012 articles were obtained through the literature searches, and the number of published articles increased annually (Figure 2A). Additionally, Citespace was used for keyword colinear analysis (Figure 2B), and the polar coordinate histogram was calculated according to the count value (Figure 2C). Analysis from the publications over the past 10 years showed that the research hotspots for traditional Chinese medicine in the treatment of RSA mainly focused on the following key words: recurrent spontaneous abortion, TCM treatment, tonifying kidneys, and promoting blood circulation, KTH, integrated traditional Chinese and Western medicine, clinical research, experience of famous doctor, prethrombotic state, progesterone, kidney deficiency and blood stasis, the damage of pre culture, and deficiency of spleen and kidney (Yang et al., 2013; Obstetrics; Subgroup et al., 2019; Cao et al., 2021; Li et al., 2021). The formula was used to show the keyword saliency map (Figure 2D), with a total of seven keywords. Finally, a clustering time line chart was constructed. The results showed that the main related research areas were divided into one of four categories: TCM treatment, USRA, KTH, and famous doctor experience (Figure 2E).

### Clinical Evidence Search Results

After excluding duplicate studies, 562 studies were examined according to their abstracts and titles, resulting in 41 articles in the final evaluation. Finally, nine studies (Liu et al., 2015; Xu et al., 2018; Feng, 2019; Feng et al., 2019; Han et al., 2019; Feng et al., 2020; He, 2020; Niu and Hao, 2020; Xu et al., 2020) were included in the present systematic review.

### Research Characteristics

Table 1 summarizes the basic information pertaining to the randomized controlled trials included. All of these trials were published in China. These studies included 1,054 URSA patients, of which 629 participants were assigned to the treatment group, and 425 participants were assigned to the control group. There was no significant difference in the baseline parameters of all trials.

**TABLE 1:** The basic characteristics of the included studies.

References	Sample size	Age (years)	Abortion time (days)	Times of abortions	Intervention time	Intervention measures	Duration of intervention	Main outcomes
He (2020)	T:60	T:30.12 ± 3.63	NR	T:3.35 ± 0.62	NR	T:KTHs (1/dose/day) + C	12 weeks	①③⑥
	C:60	C:29.67 ± 3.42		C:2.98 ± 0.56		C: dydrogesterone (10 mg, bid, po)		
Niu and Hao (2020)	T:60	T:28.76 ± 2.51	T:68.92 ± 5.12	T:4.12 ± 0.16	3 days after ovulation	T:KTHs (1/dose/day) + C	Until the 12th week of pregnancy	①②③④⑤⑥
	C:60	C:29.02 ± 2.47	C:69.13 ± 5.07	C:4.15 ± 0.23		C: dydrogesterone (10 mg, qd, po)		
Feng et al. (2020)	T:40	T:32.33 ± 4.74	NR	NR	NR	T: modified STP (1/dose/day) + C	4 weeks	①④⑥⑦
	C:40	C:30.75 ± 4.02				C: aspirin (25 mg, tid, po)		
Xu et al. (2020)	T:45	T:29.43 ± 5.42	T:60.02 ± 12.41	T:2.53 ± 0.69	After diagnosis	T:KTHs (1/dose/day) + C	Until 3 months of pregnancy	①②④⑤⑦
	C:45	C:29.14 ± 5.13	C:59.77 ± 11.36	C:2.47 ± 0.66		C: aspirin (25 mg, tid, po) + metacortandracin (5 mg, po, qn) + dydrogesterone (10 mg, bid, po)		
Han et al. (2019)	T1:86	T1:27.7 ± 2.8	NR	T1:2.81 ± 0.57	NR	T1:KTHs (1/dose/day)	Until the 12th week of pregnancy	①②④⑥⑦
	T2:87	T2:28.1 ± 2.6		T2:2.88 ± 0.53		T2:KTHs (1/dose/day) + C		
	C:88	C:27.6 ± 2.5		C:2.72 ± 0.48		C: dydrogesterone (10 mg, bid, po)		
Feng (2019)	T1:79	T1:28.6 ± 3.1	NR	T1:2.67 ± 0.47	NR	T1:KTHs (1/dose/day)	12 weeks	①②④⑦
	T2:84	T2:29.1 ± 2.5		T2:2.79 ± 0.45		T2:KTHs (1/dose/day) + C		
	C:44	C:28.2 ± 2.7		C:2.65 ± 0.44		C: dydrogesterone (10 mg, bid, po)		
Feng et al. (2019)	T:30	T:30.5 ± 2.7	T:47.6 ± 5.6	T:30.5 ± 2.7	NR	T:KTHs (1/dose/day) + C	4 weeks	①④⑤
	C:30	C:28.6 ± 2.9	C:48.3 ± 5.6	C:28.6 ± 2.9		C: dydrogesterone (10 mg, bid, po)		
Xu et al. (2018)	T:28	T:28.5 ± 3.5	T:55.3 ± 16.8	T:3.2 ± 0.6	NR	T:KTHs (1/dose/day) + C	Until the 20th week of pregnancy	①②③④⑥⑦
	C:28	C:29.3 ± 3.0	C:56.7 ± 15.4	C:3.6 ± 0.4		C:Active immunotherapy (intradermal injection every 3 weeks, two times for a course of treatment)		
Liu et al. (2015)	T:30	T:28.73 ± 4.29	NR	T:2.4 ± 0.62	3 months before planned pregnancy	T: KTHs (1/dose/day) + C	Until the 12th week of pregnancy	①③⑥
	C:30	C:30.77 ± 5.70		C:2.60 ± 0.72		C: progesterone capsule/progesterone injection (20～40 mg po/im qd)		

Note. T, trial group; C, control group; NR, not reported; ①, clinical response rate; ②, syndrome integral; ③, pregnancy outcome; ④, immune function-related outcome indicators; ⑤, coagulation function test; ⑥, reproductive hormone test; ⑦, adverse event report.

The patients in the treatment group were treated with KTHs or combined with Western medicine, while the patients in the control group were treated with Western medicine alone. Of those, six randomized controlled trials used natural progesterone, including injection, capsule, or desgesterone (Liu et al., 2015; Feng, 2019; Feng et al., 2019; Han et al., 2019; He, 2020; Niu and Hao, 2020). One study used natural progesterone in combination with other treatments (Mekinian et al., 2016), and one used active immunotherapy (Xu et al., 2018). The treatment duration ranged from 4 to 20 weeks of gestation.

### Bias Risk Assessment

**FIGURE 3:**
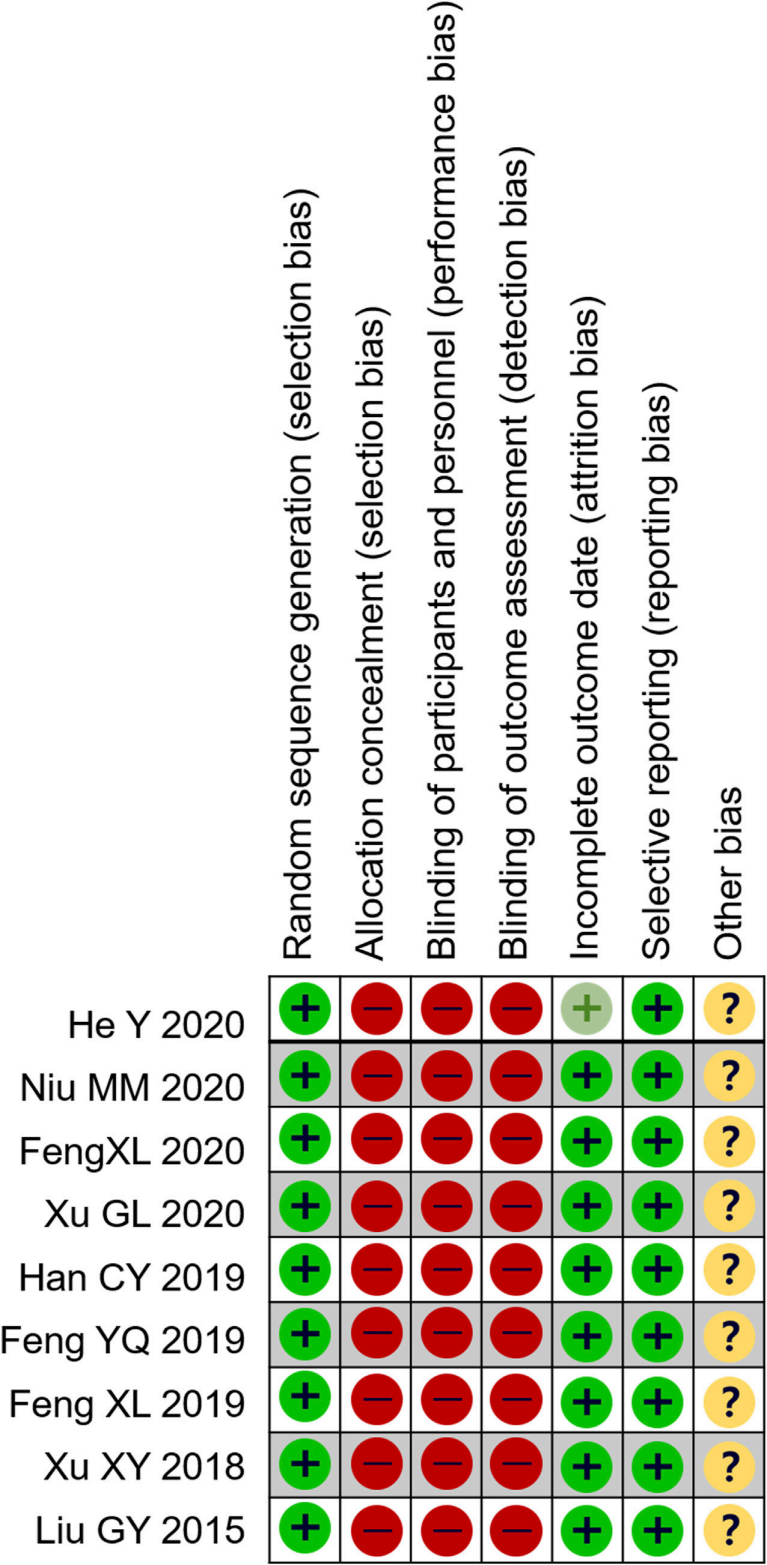
Risks of bias assessment.

Overall, the methodological quality of the included trials was poor. All nine studies were designed with two arms and were declared randomized controlled trials. None of the trials reported any details relating to the blinding of patients and researchers. No trials showed the number and reasons of dropouts. The evaluation results are shown in Figure 3.

### Outcome Analysis of Clinical Research

#### Main Outcomes

**FIGURE 4:**
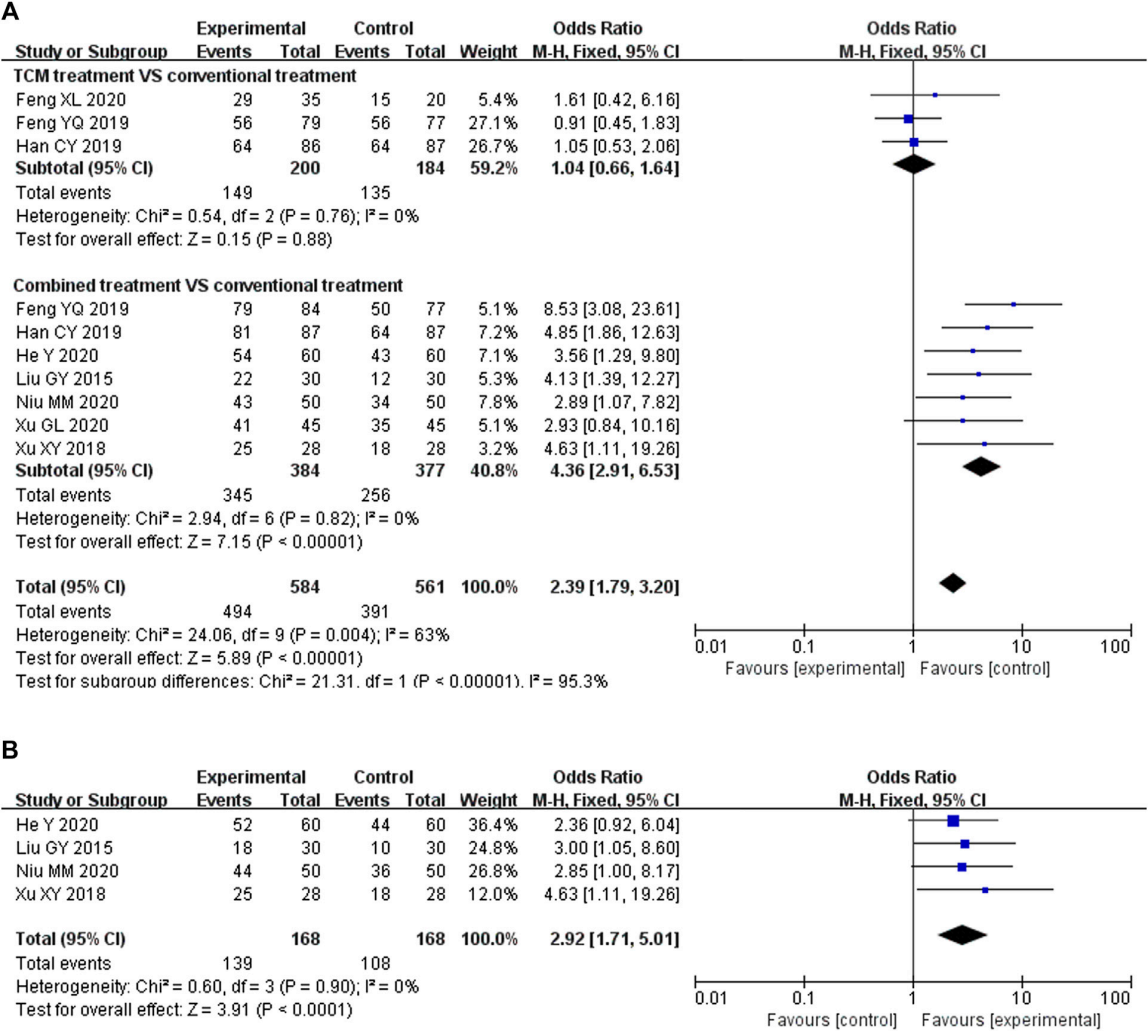
Forest plot of experimental treatment vs. control treatment for the main outcomes. **(A)** Forest plot of clinical response rate of KTHs treated URSA (combined treatment vs. conventional treatment or pure Western medicine vs. KTHs). **(B)** Forest plot of pregnancy outcomes of KTHs treated URSA.

Nine studies (Liu et al., 2015; Xu et al., 2018; Feng, 2019; Feng et al., 2019; Han et al., 2019; Feng et al., 2020; He, 2020; Niu and Hao, 2020; Xu et al., 2020) reported the clinical response rate. The pregnancy outcome was reported in four trials (Liu et al., 2015; Xu et al., 2018; He, 2020; Niu and Hao, 2020). Compared with the pure Western medicine treatment group, the pregnancy success was higher for the KTHs/KTHs with Western medicine combined group (RR: 2.92; 95% CI: 1.71–5.01: *p* < 0:01, I^2^ = 0%). Meta-analysis showed that the incidence of early pregnancy loss in the KTHs group was significantly lower than that observed in the control group (Figure 4B). Subgroup analysis (Figure 4A) showed that there was no difference in the efficacy between pure Chinese medicine and Western medicine (RR: 1.04; 95% CI: 0.66–1.64; *p* = 0.88, I^2^ = 0%), however, the combination of KTHs and modern drug therapy showed better effects (RR: 4.36; 95% CI: 2.91–6.53; *p *< 0:01, I^2^ = 0%).

#### Secondary Outcomes

**FIGURE 5:**
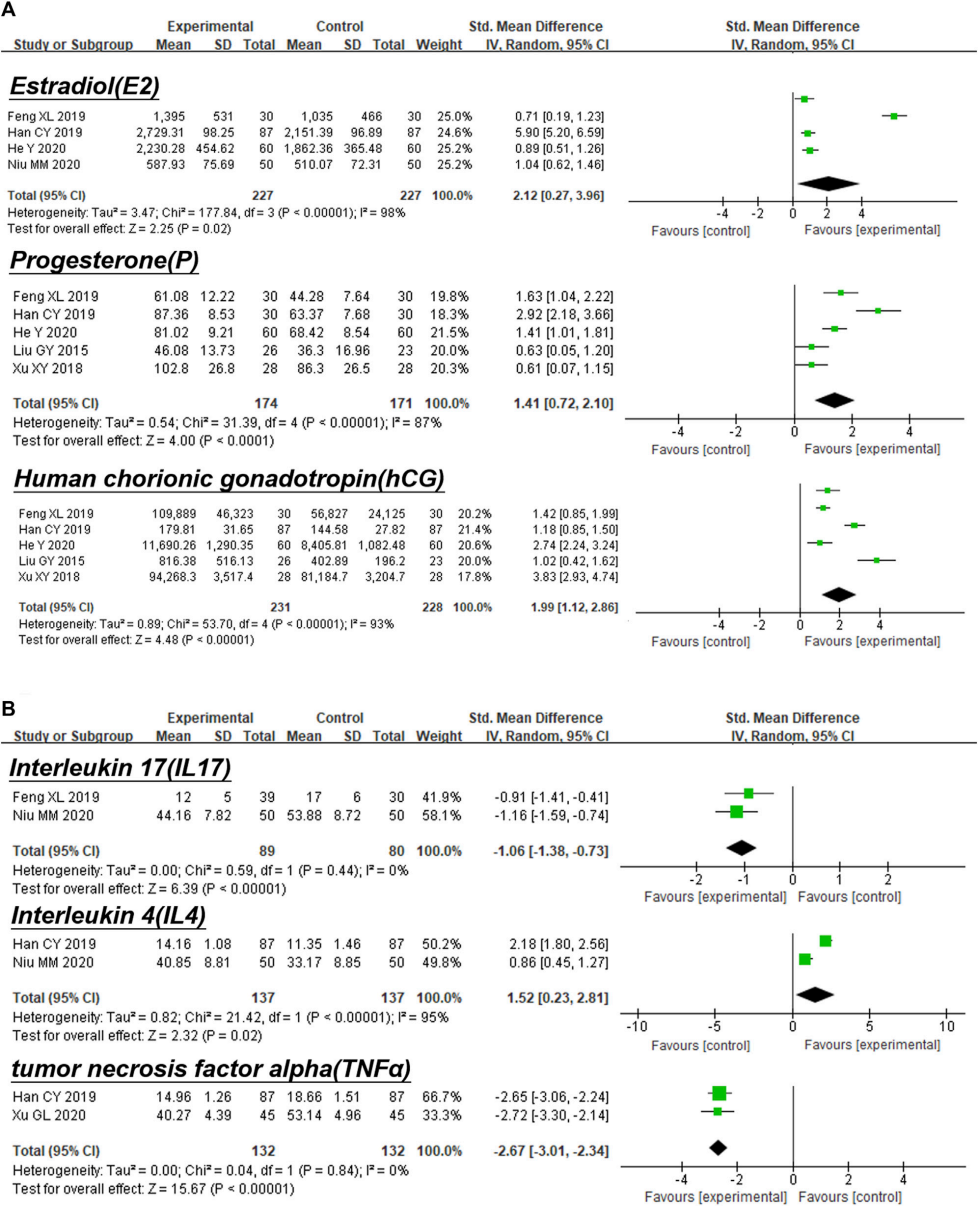
Forest plot of experimental treatment vs. control treatment in secondary outcomes. **(A)** Forest plot of hormone index. **(B)** Forest plot of serum immunological indexes.

Six of the nine studies enrolled (Liu et al., 2015; Xu et al., 2018; Han et al., 2019; Feng et al., 2020; He, 2020; Niu and Hao, 2020) reported estradiol and progestins before and after KTH treatment in the URSA patients, as detailed in Figure 5. The conclusions were consistent across the studies.

#### Estradiol

Combination therapy of KTHs and Western medicine could result in an increase in E2 compared with Western medicine alone (RR: 2.12; 95% Cl 0.27 to 3.96; *p* < 0.00001; I^2^ = 98%; random-effects model; four studies; very low-certainty evidence) (Figure 5A) (Feng, 2019; Han et al., 2019; He, 2020; Niu and Hao, 2020).

#### Progesterone

Combination therapy of KTHs and Western medicine could result in an increase in P compared with Western medicine alone (RR:1.41; 95% Cl 0.72 to 2.10; *p *< 0.00001; I2 = 87%; random-effects model; five studies; very low-certainty evidence) (Figure 5A) (Liu et al., 2015; Xu et al., 2018; Feng, 2019; Han et al., 2019; He et al., 2020).

#### Human Chorionic Gonadotropin

Combination therapy of KTHs and Western medicine could result in an increase in hCG compared with Western medicine alone (RR:1.99; 95% Cl 1.12 to 2.86; *p *< 0.00001; I^2^ = 93%; random-effects model; five studies; very low-certainty evidence) (Figure 5A) (Liu et al., 2015; Xu et al., 2018; Feng, 2019; Han et al., 2019; He et al., 2020). Seven studies (Xu et al., 2018; Feng, 2019; Feng et al., 2019; Han et al., 2019; Feng et al., 2020; Niu and Hao, 2020; Xu et al., 2020) reported changes in immune function-related outcome measures in URSA patients treated with KTHs, mainly comprising interleukin family expression and T-cell subset proportion distribution, with generally consistent expression trends after treatment, as detailed in Figure 5.

#### Interleukin 17

Combination therapy of KTHs and Western medicine could decrease the level of IL17 compared with Western medicine alone (RR: 1.06; 95% Cl −1.38 to −0.73; *p* = 0.44; I^2^ = 0%; random-effects model; two studies; very low-certainty evidence) (Figure 5B) (Feng, 2019; Niu and Hao, 2020).

#### Interleukin 4

Combination therapy of KTHs and Western medicine could result in an increase in IL4 compared with Western medicine alone (RR:1.52; 95% Cl 0.23 to 2.81; *p *< 0.0001; I^2^ = 95%; random-effects model; two studies; very low-certainty evidence) (Figure 5B) (Han et al., 2019; Niu and Hao, 2020).

#### Tumor Necrosis Factor Alpha

Combination therapy of KTHs and Western medicine could decrease the level of TNFa compared with Western medicine alone (RR: −2.67; 95% Cl −3.01 to −2.34; *p* = 0.84; I^2^ = 0%; random-effects model; two studies; very low-certainty evidence) (Figure 5B) (Han et al., 2019; Xu et al., 2020).

### Publication Bias and Sensitivity Analysis

Although the funnel plot of early pregnancy loss rate was asymmetrically distributed, Egger’s test analysis showed only marginally significant publication bias (*p* = 0.09). The sensitivity analysis of early pregnancy loss rates showed that the effect evaluation remained unchanged, indicating no strong publication bias of the combined results.

### Coagulation- and Anticoagulation-Related Outcome Indicators

Three studies (Feng et al., 2019; Niu and Hao, 2020; Xu et al., 2020) included changes in indicators related to coagulation and anticoagulation, such as PT, APTT, TT FIB, t-PA, and PAI-I in URSA patients before and after treatment with KTHs. Unfortunately, due to the large variations in detection methods, modes of intervention, and modes of comparison among the studies, a quantitative analysis of this data was not possible. The wide variation in results across the studies precludes formation of a consistent conclusion. Finally, we developed a grade assessment for the meta-analysis results based on the Cochrane recommendation (Supplementary Data Sheet S3), but the quality of clinical evidence and grade of recommendation for these indicators are of concern.

### Drug Screening and Data Intersection

**FIGURE 6:**
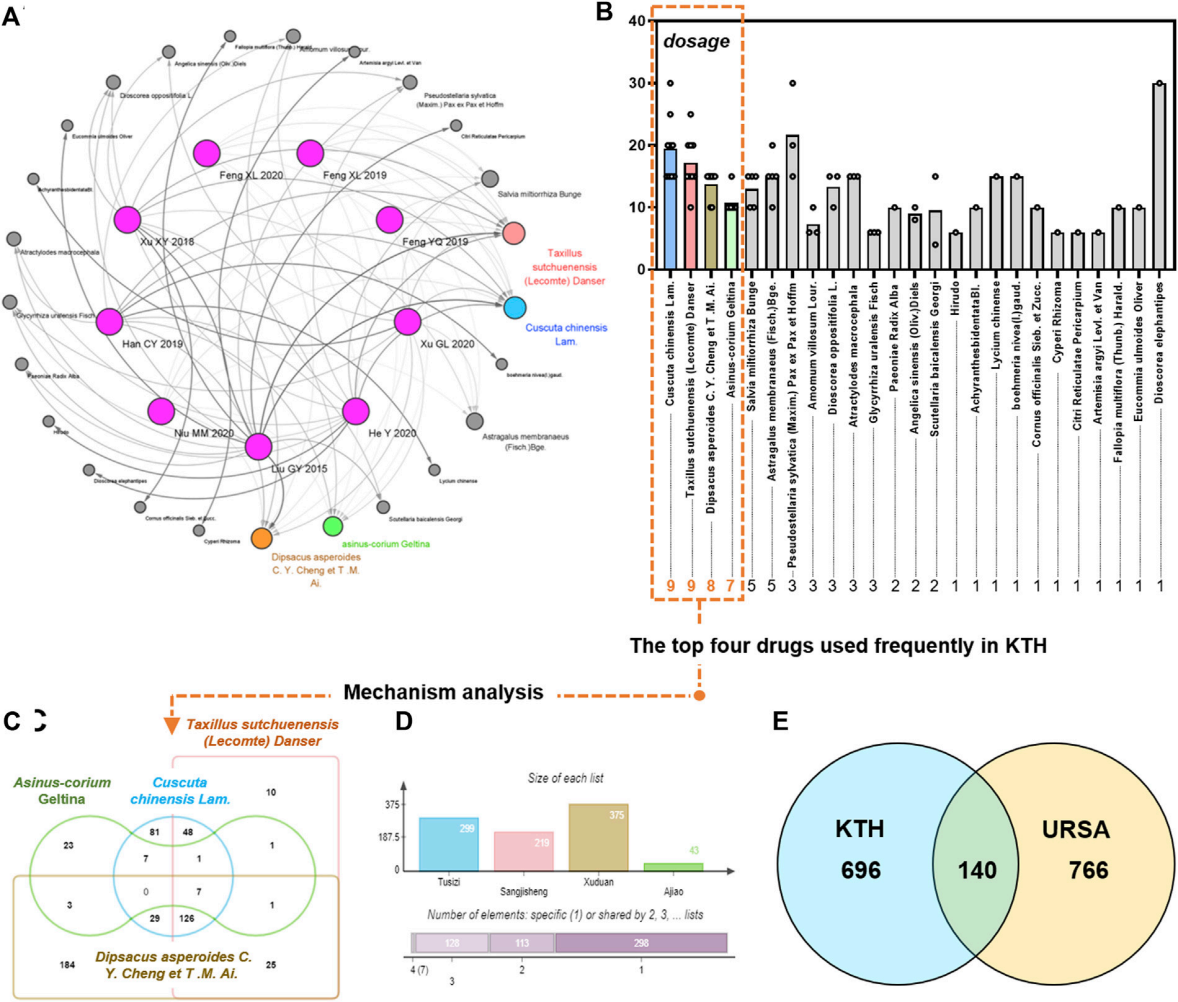
Selection of representative drugs and screening of action targets of KTHs. **(A)** Drug network. **(B)** The top drugs used frequently in KTHs. **(C)** The drug targets and URSA targets are intersected. **(D)** The ingredients of the four herbs in KTHs. **(E)** Venn diagram of drug and disease targets.

A drug network was constructed for the nine articles screened (Figure 6A). Statistics were also performed on the drug doses and frequencies used (Figure 6B). The drugs with the top four tastants of frequency, which were set at ob ≥ 30% and DL ≥ 0.18 in the tcmsp database, were selected to screen the active ingredients of the screened drugs acanthopanax, *Sambucus parasitica*, and *Cuscuta cuspida* sequestration. Additionally, the ingredients of the four herbs in KTHs were also searched in the Batman database (Figure 6D), resulting in a total of 32 potential active ingredients after removal of any duplicates from both databases. Furthermore, 546 drug targets were screened using the Swiss target prediction database with strict criteria of the species as “human” and a probability greater than 0.6. A total of 708 disease targets for URSA were obtained after dereplication. Finally, 546 drug targets and 708 disease targets were established, and a Venn diagram was drawn using the venny2.1 online software mapping tool platform, and 113 drug disease common targets were obtained after intersection of the two sets (Figure 6E).

### PPI Network Construction and Core Target Analysis

**FIGURE 7:**
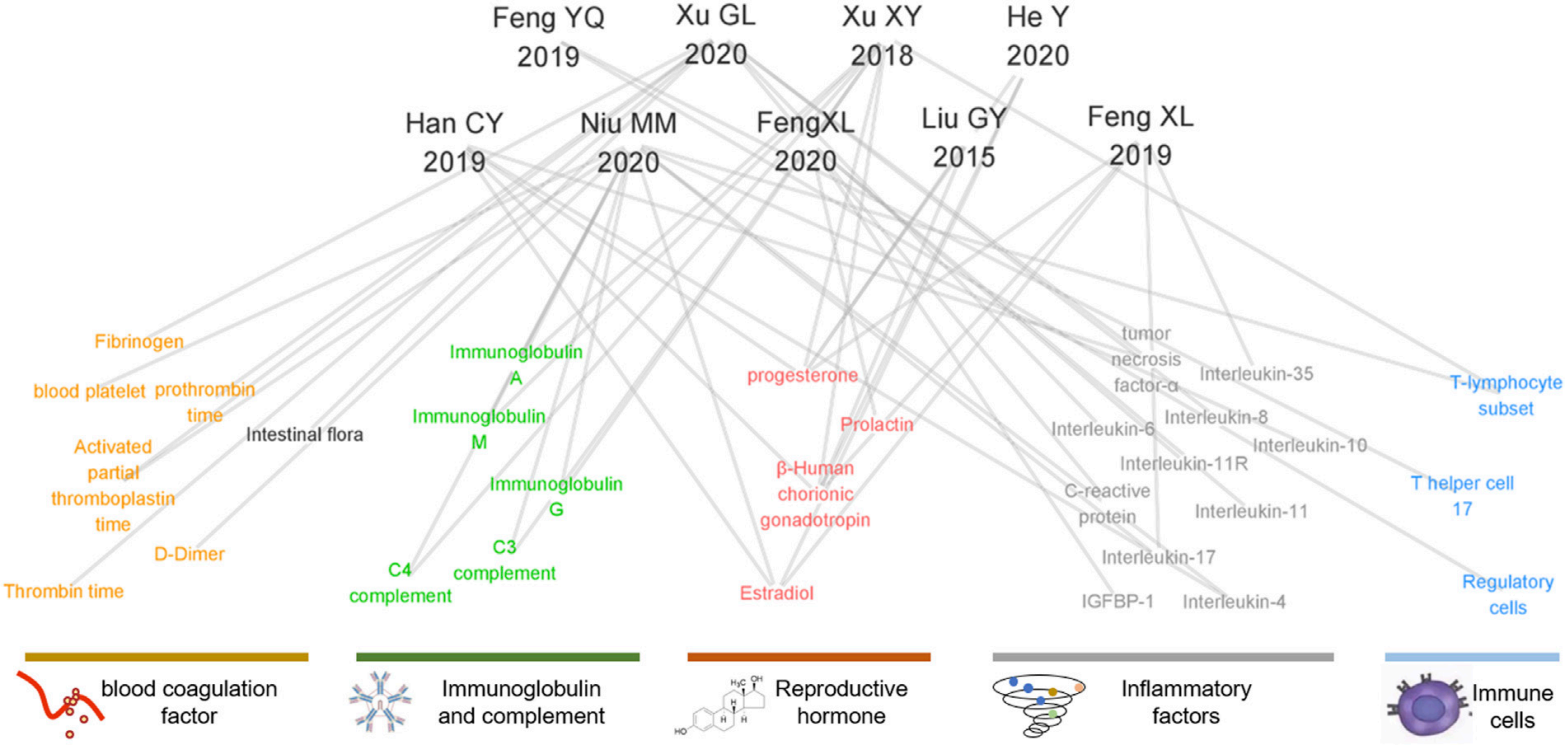
Network diagram of screening literature research indicators.

**FIGURE 8:**
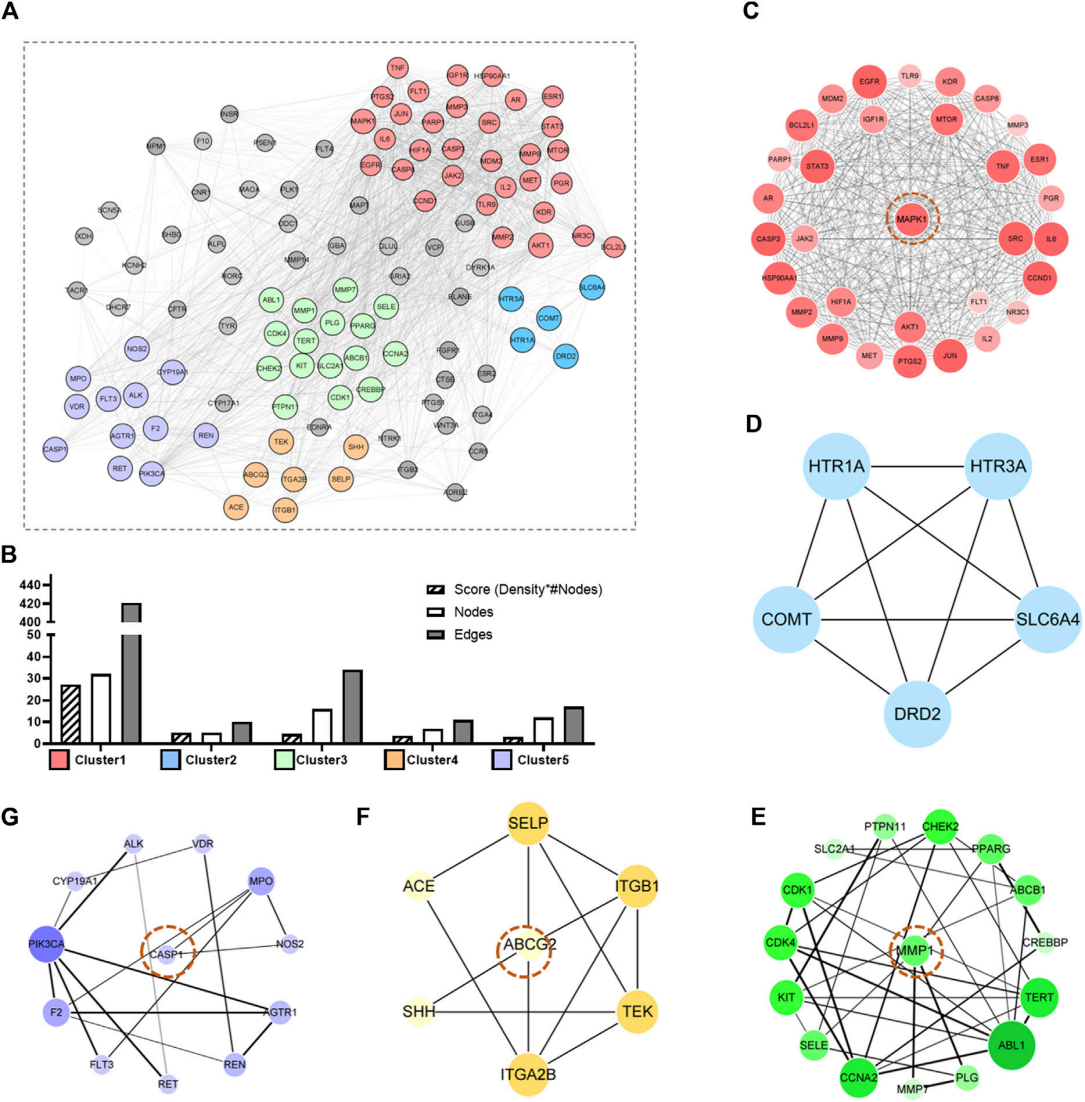
PPI analysis and core gene screening. **(A)** Protein interaction network of intersecting genes. **(B)** The top five DEGs with the score (density * # nodes), nodes, and edges of each cluster were expressed. **(C–G)** The network graph of five foundation clusters.

The 113 intersection targets were brought into the string database to construct the PPI network conditional on the species “human” and a confidence score ≥0.4. The results were imported into Cytoscape. Figure 7 shows the intersection target PPI network. The larger the node, the larger the degree value was. Next, the PPI network of URSA was further analyzed by mcode module with score cutoff = 0.2 and K core = 2. The clustering analysis was performed conditional on maximum depth = 100 and degree cutoff = 2. A total of 16 DEGs were obtained, and the top five DEGs with the highest score were taken (Figure 8B). In total, five DEGs (Figures 8C–G) and four core genes were obtained, and these five foundation clusters were scored. After screening, the four core genes obtained were mitogen-activated protein kinase (MAP1), matrix metalloprotinase1 (MMP1) (Zhang et al., 2021), ATP-binding cassette subfamily G member 2 (ABCG2), and recombinant caspase 1 (CASP1).

### Molecular Docking Verification

**FIGURE 9:**
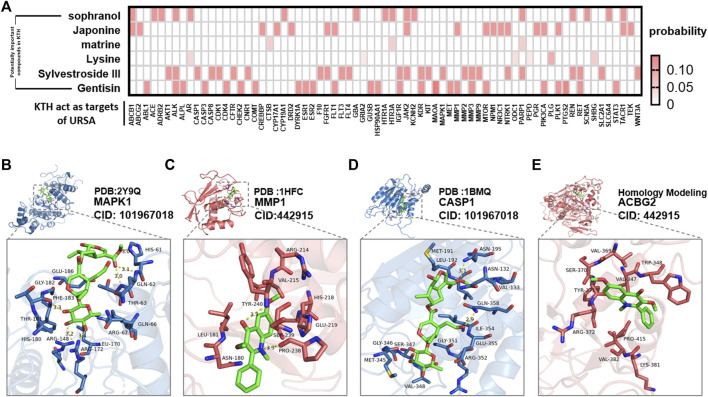
Molecular docking results of active components for KTH and URSA targets. **(A)** The heatmap of docking possibilities of major effective compounds and target proteins of KTHs. **(B–E)** The best conformation of the most effective compound and its corresponding protein receptor in KTHs.

Using different drug complexes in KTH as active compounds, the core proteins analyzed by mcode were used as targets for the molecular docking validation (Figure 9A), and the best binding compounds were selected to construct the docking map. The binding energy of Sylvestroside III small molecules with MAPK1 protein was −7.6 kcal/mol, which proved to have a good binding interaction (Figure 9B). Sylvestroside III small molecules interact with the MAPK1 protein, mainly through the formation of hydrogen bonds as well as hydrophobic forces. Six hydrogen bonds formed with ARG-148, ARG-172, THR-63, GLN-62, HIS-61, and PHE-183, respectively, with hydrogen bond lengths of 3.2, 3.4, 3.0, 3.1, 3.0, and 3.3 Å. Sylvestroside III has hydrophobic interactions with LEU-170, ARG-67, GLN-66, GLU-186, GLY-182, THR-181, and HIS-180.

Japonine small molecules, on the other hand, also bind well to the MMP1 protein, with a binding energy of −6.3 kcal/mol (Figure 9C). Avicularin small molecules interact with the MMP1 protein, mainly through the formation of hydrogen bonds as well as hydrophobic forces, resulting in seven hydrogen bonds with PRO-238 and TYR-240 with a hydrogen bond of 3.3 and 3.3 Å, respectively. Japonine has hydrophobic interactions with ASN-180, LEU-181, VAL-215, ARG-214, HIS-218, GLU-219, and SER-239. Molecular docking results confirmed the results of the network analysis relating to the effect of KTHs when treating URSA.

### Enrichment Analysis of BP and KEGG

**FIGURE 10:**
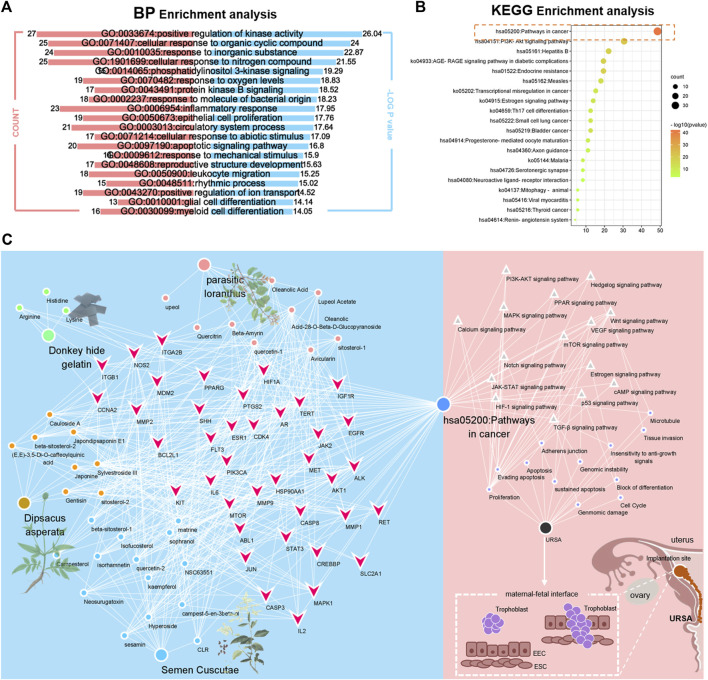
BP and KEGG enrichment analysis of URSA under the action of KTHs. **(A)** Bioaccumulation analysis of mcode core targets, the left part represents the count value, and the right part represents the log *p*-value. **(B)** Enrichment and distraction of core target KEGG based on Metascape. **(C)** Construction of drug component target pathway manifestation disease framework.

After running mcoed core clustering through Metascape, GO analysis selected biological processes for analysis. The BP results showed that the intersection gene set was enriched to 2,045 biological process pathways (Figure 10A), mainly including: positive regulation of kinase activity, cellular responses to organic cyclic compound, responses to inorganic substance, cellular responses to nitrogen compound, phosphatidylinositol 3-kinase signaling, responses to oxygen levels, protein kinase B signaling, responses to molecules of bacterial origin, inflammatory responses, epithelial cell proliferation, and others. Core genes were selected by Metascape, with parameters min overlap = 3, *p*-value cutoff = 0.01, and min enrichment = 1.5. Twenty KEGG pathways were screened out, and the results of the top 20 formed a bar graph of KEGG functional enrichment (Figure 10B). Padjust represented the significance of enrichment; the more the red the color is, the more significant. Finally, based on the core target screening and molecular docking, together with the results of BP and KEGG enrichment analyses, the 32 potentially active ingredients from the lifespan pill were the inputs with the 113 drug disease cotargets into Cytoscape software to remove isolated ingredients with no intersection with targets. A network diagram of “drug ingredient target disease” interactions was drawn (Figure 10C). Using the score of 30 potentially active ingredients, and the average of the degrees as the screening criterion, we selected six compounds including Sylvestroside III, Japonine, sophranol, Gentisin, Lysine, and Matrine as core ingredients that were either large at the network nodes or showed high binding to protein targets (The screening process and criteria are detailed in Supplementary Data Sheet S4). The most important active compounds that were selected are listed in Table 2.

**TABLE 2:** Core components and targets of KTHs.

Pubchem CID	Key components	Molecular structure	Weight g/mol	Possible target of URSA
101967018	Sylvestroside III	C_27_H_36_O_14_	584.60	AKT1, ALK, CASP1, CASP8, CDK1, CFTR, CNR1, F10, FGFR1, FLT3, FLT4, IGF1R, JAK2, KDR, KIT, MAPK1, MMP2, MMP3, MMP9, NTRK1, PARP1, REN, RET, SLC2A1, and WNT3A
442915	Japonine	C_18_H_17_NO_3_	295.30	ABCB1, ABCG2, CREBBP, CYP17A1, DRD2, FGFR1, FLT1, JAK2, MMP1, MTOR, NPM1, NR3C1, NTRK1, PGR, PIK3CA, PLK1, TACR1, and TEK
12442899	sophranol	C_15_H_24_N_2_O_2_	264.36	ABCB1, ACE, ADRB2, ALK, AR, CYP19A1, GBA, HTR1A, HTR3A, JAK2, KCNH2, PARP1, REN, SCN5A, SLC6A4, and TACR1
5281636	Gentisin	C_14_H_10_O_5_	258.23	MAOA, PTGS2, ABL1, ALK, ALPL, CASP3, CDK4, CHEK2, COMT, DYRK1A, ESR1, ESR2, FLT1, GUSB, HSP90AA1, IGF1R, KDR, MET, MMP1, MMP3, MTOR, PIK3CA, PLK1, RET, and STAT3
5962	Lysine	C_6_H_14_N_2_O_2_	146.19	PLG, SHBG, AR, ODC1, GRIA2, and PEPD
91466	Matrine	C_15_H_24_N_2O_	248.36	CTSB, HTR3A, and PARP1

## Discussion

At present, there is a lack of targeted diagnostic and treatment options for URSA, all of which provide suboptimal clinical outcomes (Pierce, 1983; Youssef et al., 2019; Li et al., 2020). The question on how to find better interventions to achieve good pregnancy outcomes for more URSA patients is currently a hot topic and a challenging point in the field of reproductive medicine.

Although progesterone was used as the primary method of treatment in most of the studies included in this research, it has limited clinical efficacy and can cause side effects such as dizziness, nausea, and vomiting (Yan et al., 2013; Coomarasamy et al., 2020a; Coomarasamy et al., 2020b). Potential risks include allergies, infectious diseases, and bleeding, as well as an ever-increasing financial burden on individuals and society. In the future, new methods for treating URSA will be necessary. As a supplement, traditional Chinese medicine, based on four main diagnostic methods, which regulate the human body as a whole using an inspection method, auscultation, olfaction method, and pulse diagnosis method, looks promising (Zhou et al., 2007). In TCM, KTHs are considered as a treatment, which pays more attention to the syndrome of patients and infers localized treatment from the whole, which may be the result of multichannel and multitarget approaches. However, modern medicine emphasizes the specific etiological treatment affecting URSA, such as focusing in on improving the uterine blood supply and endometrial immune status of the patient. Although KTH has a complex composition and a presently unclear mechanism of action, its comprehensive effects on diseases, especially on health care and prevention, are temporarily irreplaceable by modern medicine (Li et al., 2016; Luo et al., 2012; Liu et al., 2009).

**FIGURE 11:**
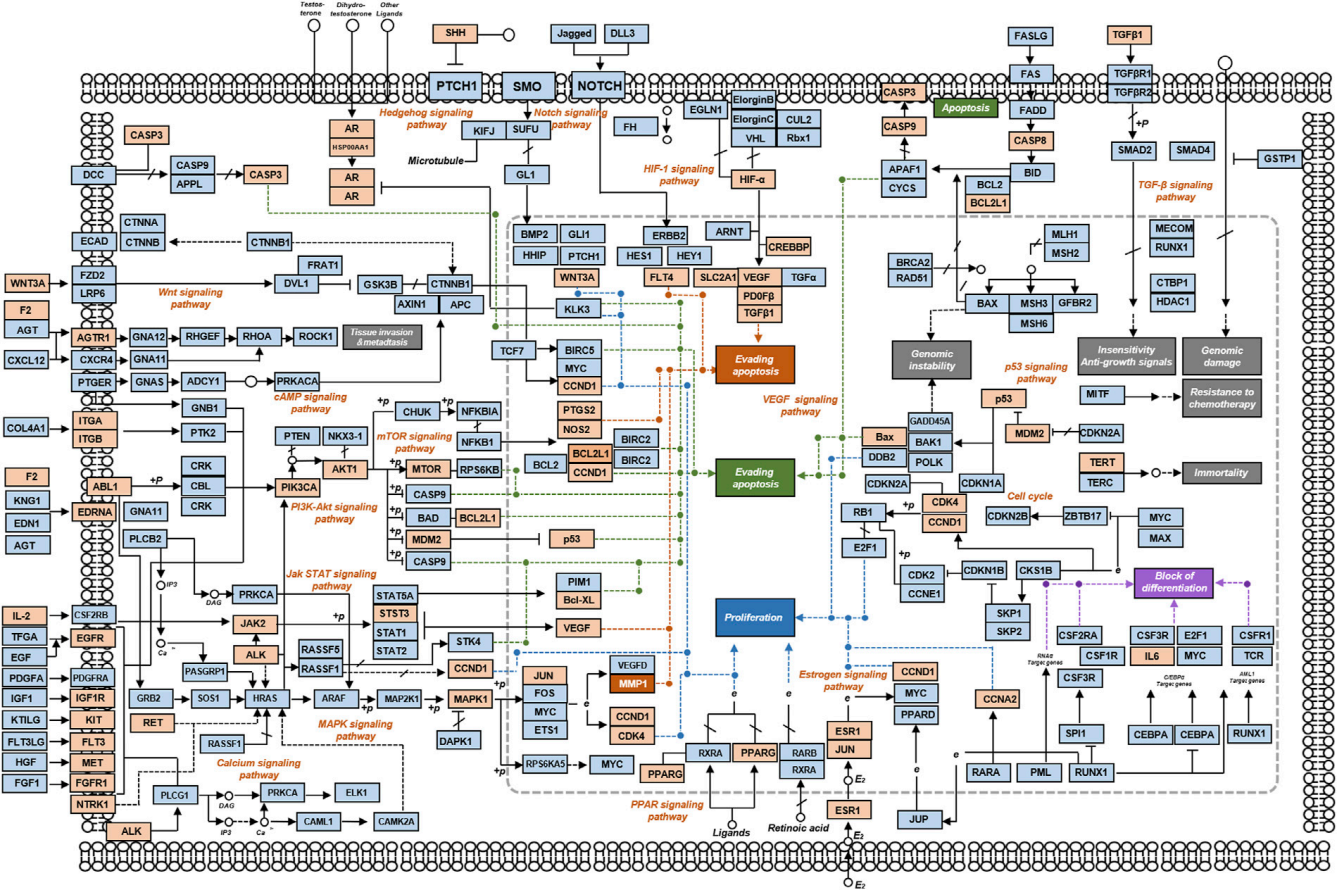
Tumor signaling pathways interact with a variety of signaling pathways, which are closely related to embryo implantation. Orange represents the core target of KTHs.

**FIGURE 12:**
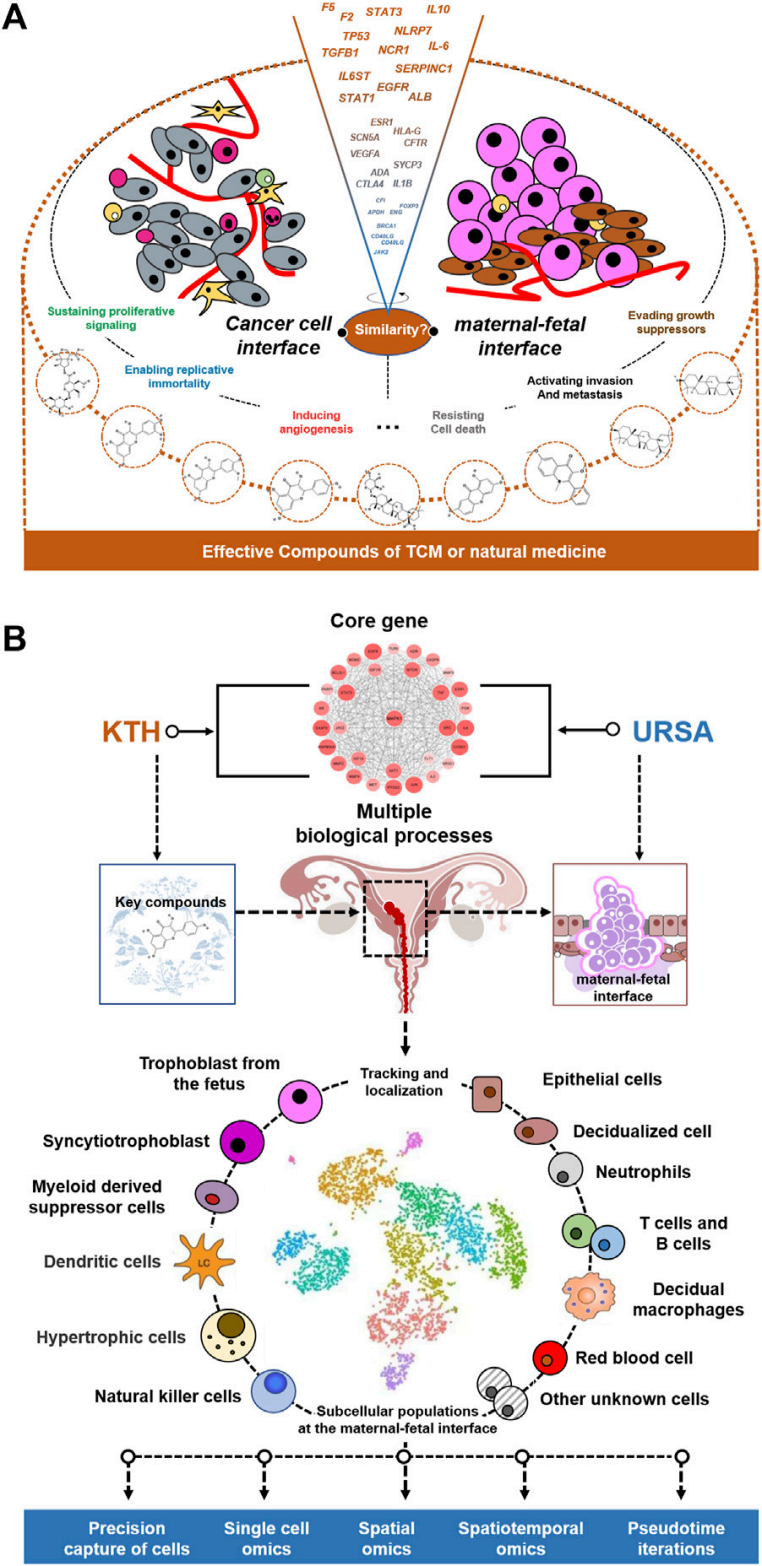
Possible treatment R&D ideas in the future. **(A)** Similarities between malignant tumor and embryo implantation: a new entry point of USRA treatment mechanism. **(B)** The cross- core genes of KTHs and URSA are used to guide the results of single cells.

Oncology is an important crossover area for reproduction. Based on the evidence from this meta-analysis, a network pharmacological approach was used to investigate which targets are important, and how these targets and signaling pathways play a role in RSA. Since the concept of an embryonal origin of cancer was proposed in 1982 by Lobstein, the resemblance between the biological behaviors of embryo implantation and tumor invasion and metastasis has been increasingly recognized (Figure 11) (Murray and Lessey, 1999; Perry et al., 2009). Especially given that “pseudo malignant” blastocyst trophoblast cells and malignant cells (Soundararajan and Rao, 2004; Fest et al., 2008) exhibit defects in cell proliferation and differentiation, invasion signaling pathways, vascular erosion, and neovascularization. There are additional striking similarities in many aspects of both processes, such as immune escape and apoptosis. The most fundamental biological process during embryo implantation is the invasive properties of trophoblast cells (Hanahan and Weinberg, 2011), which is regulated by a network of extracellular matrix, matrix degrading enzyme, cell adhesion molecules, and growth factors (Figure 12A).

The results of this study suggest that the main signaling pathway of KTHs when used to treat URSA is a cancer signaling pathway, which confirms our conjecture. This signaling pathway dominates many biological processes. Therefore, we identified several main biological processes and related targets, which are regulated by KTH active components, and compared this biological process between embryo implantation and cancer progression, as shown in Table 3, in order to obtain more insights and beneficial information.

**TABLE 3:** The main biological processes of cancer progression and embryo implantation, and the targets of related components in traditional Chinese medicine.

Cell biological processes	The significance in embryonic development	The significance in cancer progression	The related genes obtained in this study	Components of KTHs
Regulation of epithelial cell proliferation	Promote the uterus into the receptive state, decidualization of uterine stromal cells and the occurrence of placenta (Zhu et al., 2019)	It can make tumor proliferate rapidly, compress tissue, and promote angiogenesis (Zhong et al., 2020)	AR, TNF, XDH, PPARG, PGR, VDR, STAT3, JUN, MTOR, EGFR, KDR, AKT1, SHH, ITGB3, SCN5A, HIF1A, AGTR1, ITGA4, GLUL, FLT1, FGFR1, TEK, FLT4, WNT3A, and CCND1	(a), (b), (d), €, and (f)
Epithelial cell migration	It can promote the mutual recognition and interaction between blastotrophoblast cells and endometrial cells, and promote the balance of maternal–fetal interface (Zhu et al., 2019)	Cell migration plays an important role in the early occurrence and development of various cancers, especially before primary tumor cells develop into invasive lesions (Hsieh and Wu, 2020)	PTGS2, TNF, PPARG, PTPN11, JUN, MTOR, PIK3CA, SRC, KDR, MMP9, MET, AKT1, ITGB3, HIF1A, ITGB1, GLUL, ABL1, FGFR1, TEK, FLT4, and KIT	(b), (e), and (f)
Regulation of tissue remodeling	Embryo implantation and development involve degradation and remodeling of extracellular matrix, placental villous vasculogenesis, and reconstruction of uterine spiral artery (Zhang et al., 2021)	Tumor destroys normal tissue and makes it remodel to change its original biological function (Zhong et al., 2020)	PLG, IL2, VDR, MDM2, MMP2, MMP14, EGFR, SRC, ITGB3, HIF1A, ADRB2, ACE, IL6, and FLT4	(a), (d), and (e)
Response to oxidative stress	Excessive ROS can cause mitochondrial damage, DNA damage, lipid peroxidation, and even cell apoptosis in embryos, and then lead to embryonic development arrest (Ivanov et al., 2021)	High level and long-term oxidative stress can directly damage tissues through this redox system, and also lead to oxidative modification of amino acid residues and DNA mutation, thus promoting the occurrence of tumor (Shrivastava et al., 2021)	PTGS2, TNF, PTGS1, MDM2, JUN, MMP3, MMP2, MMP14, EGFR, MPO, SRC, CDK1, MMP9, MET, AKT1, MAPT, PARP1, HIF1A, JAK2, CASP3, IL6, ABL1, CCNA2, MAPK1, and PSEN1	(a), (b), (c), (e), and (f)
Regulation of mitochondrion organization	In the process of embryo implantation, the number, distribution, and activity of mitochondria are regulated strictly and orderly, and affect the embryo implantation potential at the same time (Moher et al., 2009)	The malignant phenotypes of tumor cells, such as unlimited proliferation, abnormal metabolism, inhibition of apoptosis, strong invasion, and easy metastasis, are also closely related to mitochondrial dysfunction (Roth et al., 2020; Suldina et al., 2018)	BCL2L1, KDR, MMP9, AKT1, MAPT, HIF1A, GBA, and CASP8	(a), (e), and (f)
Response to lipopolysaccharides	Inflammation and infection result in LPS affecting decidual differentiation through toll-like receptor 4, which leads to stress injury and thrombosis of trophoblast (Guo et al., 2019)	LPS can promote tumor survival by activating upregulated inflammatory signaling pathway, and can also increase the expression of adhesion factors of tumor cells to endothelial cells by activating neutrophils (Shetab et al., 2019)	NOS2, PTGS2, TNF, CNR1, MPO, SRC, AKT1, CCR5, REN, JAK2, ELANE, SELE, SELP, CASP3, CASP8, CASP1, IL6, CDK4, ALPL, ABL1, COMT, and MAPK1	(a), (b), (e), and (f)
Regulation of autophagy	Autophagy can affect embryo delayed implantation, abnormal decidua, and reduce the expression of autophagy in endometrial receptive period to ensure the success of embryo implantation (Su et al., 2020)	Moderate autophagy can make the damaged tumor cells survive, while excessive autophagy can accelerate the death of tumor cells (Wang et al., 2019)	STAT3, MTOR, PIK3CA, KDR, MET, AKT1, MAPT, HIF1A, GBA, ADRB2, CASP3, and ABL1	(a), (b), (e), and (f)
Regulation of apoptotic signaling pathway	Proapoptotic factors and antiapoptotic factors play a key role in regulating the survival and apoptosis of embryonic cells, and the balance between them determines the survival or death of embryos (Zhang et al., 2021)	Imbalance in the ratio of proliferation and apoptosis of tumor cells is the key factor in tumorigenesis and progression (Mohamed et al., 2017)	AR, PTGS2, TNF, TERT, MDM2, BCL2L1, SRC, MMP9, AKT1, PARP1, HIF1A, JAK2, CASP8, RET, FGFR1, and PSEN1	(a), (b), (c), (d), (e), and (f)
Regulation of inflammatory response	The balance of pro-inflammatory factors and anti-inflammatory factors promotes endometrial receptivity, and appropriate inflammatory environment promotes embryo implantation and pregnancy maintenance (Quirke et al., 2021)	The infiltration of inflammatory cells and the production of ROS are necessary and sufficient conditions to accelerate the carcinogenesis (Rossi et al., 2021)	NOS2, PTGS2, TNF, IL2, ESR1, CYP19A1, PPARG, CNR1, F2, TLR9, STAT3, MMP3, EGFR, MMP9, GBA, JAK2, ELANE, SELE, CASP1, AGTR1, IL6, and TEK	(a), (b), (e), and (f)
Regulation of angiogenesis	On the basis of the original blood vessels, the formation of blood vessels through the process of endothelial cell proliferation, and migration is conducive to the development and infiltration of embryonic cells (Barrientos et al., 2013)	It can promote the secretion of tumor cells, promote angiogenic factors, promote the proliferation of endothelial cells, and chemotaxis the migration of endothelial cells (Li et al., 2019)	PTGS2, TNF, TERT, PPARG, STAT3, KDR, HIF1A, ITGB1, AGTR1, IL6, GLUL, FLT1, ABL1, and TEK	(b), (e), and (f)
Epithelial–mesenchymal transition, EMT	It can make endometrial epithelial and stromal cells more invasive and mobile, promote embryonic organ formation, embryonic differentiation, and nervous system differentiation (Ran et al., 2020)	EMT plays an important role in the invasion and metastasis of tumor *in situ* and the formation of new metastasis. It is an important way of invasion and metastasis of epithelial cell carcinoma, which accounts for more than 90% of malignant tumors in adults (Thiery, 2002)	MMP2, MMP7, NOS2, MET, CDK1, CYP19A1, SHH, JAK2, CCNA2, MMP1, MMP3, MDM2, EGFR, ABL1, CHEK2, PGR, RET, ABCB1, STAT3, IL6, CASP3, VDR, ABCG2, PIK3CA, IGF1R, HIF1A, TLR9, JUN, AR, TNF, ITGB1, KIT, F2, PTGS2, CCND1, ESR1, CDK4, SLC2A1, FLT1, TERT, SRC, REN, PTPN11, ALK, CASP8, PPARG, NR3C1, MTOR, DRD2, AGTR1, HSP90AA1, PARP1, KDR, BCL2L1, and MMP9	(a), (b), (c), (d), (e), and (f)

Note. The main active components in KTHs: (a) Sophranol; (b) Japonine; (c) Matrine; (d) Lysine; (e) Sylvestroside III; and (f) Gentisin.

In conclusion, tumor cells and early embryos show similar mechanisms to each other, which gives us enlightenment as to how the microenvironment of embryo growth can guide and activate the potential of tumor development and reverse its phenotype. The process of embryonic development and differentiation is almost a reversion of tumor differentiation. Further study on the regulation mechanism and important regulation steps during embryonic development can provide important indicators and cues for the study of tumor differentiation reversal.

The combination of embryo implantation and tumor invasion and metastasis not only helps understand the internal mechanism of complex life phenomena but also may provide new concepts for clinical treatment. Antitumor invasion and metastasis therapy may ultimately find the answer from embryo implantation, and repeated embryo implantation failure or repeated abortion may also provide new evidence-based answers toward understanding and treating tumors.

## Limitation and Outlook

Although we have undertaken a comprehensive analysis and evaluation of all of the published studies, this research still has some limitations, which are worthy of recognition. The quality of the included studies was low due primarily to unclear allocation concealment, selective bias, consumption bias, and the blinding methods, or lack of thereof. The one major limitation within all of the included studies was that they were all conducted in China and have major methodological flaws, which greatly reduced the reliability and validity of study results as a whole.

The approach taken helps map cell types, cell subpopulations, and even cell states in a spatial context. The similarity of pregnancy maintenance to cancer invasion allows us to recognize the high complexity of subcellular species and spatial architecture at the maternal–fetal interface (Srivatsan et al., 2021). This complexity is often accompanied by spatiotemporal dynamic changes, so the study of KTHs for URSA cannot be satisfied by employing traditional single *ex vivo* experiments with animal experiments (Figure 12B). Therefore, how KTHs could map the cell types, cell subsets, and even cell states and gene expression profiles of each class of cells regulated at the maternal–fetal interface of URSA patients in a spatial context, is a very urgent matter, which provides an important area of future research.

It is widely known that single-cell RNA sequencing (scRNA-seq) identifies cell subpopulations within tissue but does not capture their spatial distribution nor reveal local networks of intercellular communication acting *in situ*. Fortunately, a suite of recently developed techniques that localizes RNA within tissues, including multiplexed *in situ* hybridization and *in situ* sequencing (here defined as high-plex RNA imaging) and spatial barcoding, could help address this issue (Longo et al., 2021). We believe that this method of single-cell sequencing, combined with spatial transcription, will bring about a more profound insight into how KTHs acts on URSA.

## Conclusion

In conclusion, the TCM-based KTH formula provides good efficacy and safety for the treatment of URSA, and has great potential. According to the results of the network pharmacology analysis, we predicted that the main pathway of the KTH effective component in URSA is a tumor signaling pathway. With the in-depth study of the mechanisms of tumor formation, as well as single-cell spatial transcriptome in the future, will bring forth breakthroughs in the pathogenesis research and treatment of USRA.

## Data Availability

The datasets presented in this study can be found in online repositories. The names of the repository/repositories and accession number(s) can be found in the article/Supplementary Material.
