# Supplementary material for: A Potential Mechanism of Kidney-Tonifying Herbs Treating Unexplained Recurrent Spontaneous Abortion: Clinical Evidence From the Homogeneity of Embryo Implantation and Tumor Invasion
Source: Front Pharmacol. 2022 Jan 26;12:775245. doi: 10.3389/fphar.2021.775245 (PMC8826263; doi:10.3389/fphar.2021.775245)
Supplement: Supplementary file 1 [file DataSheet4.DOCX]

# SUPPLEMENTARY MATERIAL 4

## Core compound screening modalities and results

In the process of screening the most important biological small molecules of KTH, we need to consider whether these components conform to the Lipinski 5 rule. In addition to the degree of degree ranking in network analysis. In addition, we need to consider whether these active compounds are present in a wide range of herbs（For example quercetin, which exists in 327 drugs, is inappropriate to regard as the most important bioactive molecule for KTH treatment）.

**TABLE S2⏐Screening results of core compounds**

| **Ingredient name** | **Degree** | **Pubchem CID** | **HERB ID** | **Molecular Structure** | **MH**  **(g/mol)** | **Quantity of TCM** | **LogP** | **Hdon** | **Hacc** | **Rbon** |
| --- | --- | --- | --- | --- | --- | --- | --- | --- | --- | --- |
| Sylvestroside III | 31 | 101967018 | HBIN045208 | C27H36O14 | 584.60 | 2 | -1 | 5 | 14 | 11 |
| Quercetin | 28 | 5280343 | HBIN041495 | C15H10O7 | 302.23 | 341 | 1.5 | 5 | 7 | 1 |
| Isorhamnetin | 28 | 5281654 | HBIN031114 | C16H12O7 | 316.26 | 80 | 1.9 | 4 | 7 | 2 |
| Kaempferol | 28 | 5280863 | HBIN031753 | C15H10O6 | 286.24 | 248 | 1.9 | 4 | 6 | 1 |
| Gentisin | 28 | 5281636 | HBIN027522 | C14H10O5 | 258.23 | 12 | 2.8 | 2 | 5 | 1 |
| Cauloside A | 26 | 441928 | HBIN019980 | C35H56O8 | 604.8 | 11 | 4.7 | 5 | 8 | 4 |
| Japonine | 21 | 442915 | HBIN031441 | C18H17NO3 | 295.3 | 4 | 3.5 | 0 | 4 | 3 |
| Sophranol | 19 | 12442899 | HBIN044399 | C15H24N2O2 | 264.36 | 2 | 0.5 | 1 | 3 | 0 |
| Oleanolic Acid | 18 | 10494 | HBIN037936 | C30H48O3 | 456.7 | 203 | 7.5 | 2 | 3 | 1 |
| Beta-Amyrin | 16 | 73145 | HBIN017966 | C30H50O | 426.7 | 71 | 9.2 | 1 | 1 | 0 |
| NSC63551 | 16 | 5356634 | HBIN037512 | C29H48O | 412.7 | 3 | 9 | 0 | 1 | 5 |
| Oleanolic Acid-28-O-Beta-D-Glucopyranoside | 14 | 14189384 | HBIN037943 | C36H58O8 | 618.8 | 35 | 5.7 | 5 | 8 | 4 |
| Sitosterol | 14 | 222284 | HBIN044158 | C29H50O | 414.7 | 179 | 9.3 | 1 | 1 | 6 |
| Beta-sitosterol |  |  | HBIN018278 | C29H50O | 414.7 | 567 | 9.3 | 1 | 1 | 6 |
| CLR | 14 | 131698629 | HBIN021150 | C38H59BF2N3O3P | 685.7 | 32 | 0 | 0 | 6 | 24 |
| (E,E)-3,5-Di-O-caffeoylquinic acid | 14 | 6474310 | HBIN024871 | C25H24O12 | 516.45 | 1 | 1.5 | 7 | 12 | 9 |
| Isofucosterol | 13 | 5281326 | HBIN030752 | C29H48O | 412.7 | 6 | 8.9 | 1 | 1 | 5 |
| Neosurugatoxin | 9 | 46173822 | HBIN036737 | C30H34BrN5O15 | 784.5 | 3 | -5.6 | 12 | 7 | 5 |
| Campest-5-en-3beta-ol | 8 | 173183 | HBIN019471 | C28H48O | 400.7 | 14 | 8.8 | 1 | 1 | 5 |
| Campesterol | 8 | 173183 | HBIN019475 | C28H48O | 400.6 | 83 | 8.8 | 1 | 1 | 5 |
| Lysine | 7 | 5962 | HBIN034051 | C6H14N2O2 | 146.19 | 11 | -3 | 3 | 4 | 5 |
| Avicularin | 6 | 5490064 | HBIN017408 | C20H18O11 | 434.3 | 24 | 1 | 7 | 11 | 4 |
| Matrine | 6 | 91466 | HBIN034558 | C15H24N2O | 248.36 | 24 | 1.6 | 0 | 2 | 0 |
| Hyperoside | 6 | 5281643 | HBIN029837 | C21H20O12 | 464.4 | 38 | 0.4 | 8 | 12 | 4 |
| Japondipsaponin E1 | 6 | 11968646 | HBIN031428 | C59H96O26 | 1221.38 | 3 | -5.95 | 14 | 26 | 15 |
| Lupeol Acetate | 5 | 92157 | HBIN033753 | C32H52O2 | 468.8 | 42 | 10.4 | 0 | 2 | 4 |
| Quercitrin | 5 | 5280459 | HBIN041726 | C21H20O11 | 448.4 | 77 | 0.9 | 7 | 11 | 3 |
| Histidine | 3 | 6274 | HBIN029439 | C6H9N3O2 | 155.15 | 17 | -3.2 | 3 | 4 | 3 |
| Arginine | 2 | 6322 | HBIN016720 | C6H14N4O2 | 174.2 | 15 | -4.2 | 4 | 4 | 5 |
| Sesamin | 2 | 72307 | HBIN043798 | C20H18O6 | 354.4 | 34 | 2.7 | 0 | 6 | 2 |

**Note: Yellow:** Widely occurring compounds whose degree of activity is difficult to assess, **Gray:** drugs that do not comply with the libinsky rule; **Green:** potentially important kth **compoundsLibinsky 5 rule :**a molecule weight (MW) < 500, number of hydrogen bond donors (Hdon) ≤ 5, number of hydrogen bond acceptors (Hacc) ≤ 10, lipid–water partition coefficient (LogP) ≤5 and number of rotatable bonds (Rbon)≤ 10

## data of compoundsinpubchem

**TABLE S3⏐Screening results of core compounds**

| **Coumpunds** | **CID** | **URL link** |
| --- | --- | --- |
| Sylvestroside III  101967018 | **Pubchem** | https://pubchem.ncbi.nlm.nih.gov/compound/101967018 |
|  | **HERB** | http://herb.ac.cn/Detail/?v=HBIN045208&label=Ingredient |
| Quercetin  5280343 | **Pubchem** | https://pubchem.ncbi.nlm.nih.gov/compound/5280343 |
|  | **HERB** | http://herb.ac.cn/Detail/?v=HBIN041495&label=Ingredient |
| Isorhamnetin  5281654 | **Pubchem** | https://pubchem.ncbi.nlm.nih.gov/compound/5281654 |
|  | **HERB** | http://herb.ac.cn/Detail/?v=HBIN031114&label=Ingredient |
| Kaempferol  5280863 | **Pubchem** | https://pubchem.ncbi.nlm.nih.gov/compound/5280863 |
|  | **HERB** | http://herb.ac.cn/Detail/?v=HBIN031753&label=Ingredient |
| Gentisin  5281636 | **Pubchem** | https://pubchem.ncbi.nlm.nih.gov/compound/5281636 |
|  | **HERB** | http://herb.ac.cn/Detail/?v=HBIN027522&label=Ingredient |
| Cauloside A  441928 | **Pubchem** | https://pubchem.ncbi.nlm.nih.gov/compound/441928 |
|  | **HERB** | http://herb.ac.cn/Detail/?v=HBIN019980&label=Ingredient |
| Japonine  442915 | **Pubchem** | https://pubchem.ncbi.nlm.nih.gov/compound/442915 |
|  | **HERB** | http://herb.ac.cn/Detail/?v=HBIN031441&label=Ingredient |
| Sophranol  12442899 | **Pubchem** | https://pubchem.ncbi.nlm.nih.gov/compound/12442899 |
|  | **HERB** | http://herb.ac.cn/Detail/?v=HBIN044399&label=Ingredient |
| Oleanolic Acid  10494 | **Pubchem** | https://pubchem.ncbi.nlm.nih.gov/compound/10494 |
|  | **HERB** | http://herb.ac.cn/Detail/?v=HBIN037936&label=Ingredient |
| Beta-Amyrin  73145 | **Pubchem** | https://pubchem.ncbi.nlm.nih.gov/compound/73145 |
|  | **HERB** | http://herb.ac.cn/Detail/?v=HBIN017966&label=Ingredient |
| NSC63551  5356634 | **Pubchem** | https://pubchem.ncbi.nlm.nih.gov/compound/5356634 |
|  | **HERB** | http://herb.ac.cn/Detail/?v=HBIN037512&label=Ingredient |
| Oleanolic Acid-28-O-Beta-D-Glucopyranoside  14189384 | **Pubchem** | https://pubchem.ncbi.nlm.nih.gov/compound/14189384 |
|  | **HERB** | http://herb.ac.cn/Detail/?v=HBIN037943&label=Ingredient |
| Sitosterol  222284 | **Pubchem** | https://pubchem.ncbi.nlm.nih.gov/compound/222284 |
|  | **HERB** | http://herb.ac.cn/Detail/?v=HBIN044158&label=Ingredient |
| Beta-sitosterol  222284 | **Pubchem** | https://pubchem.ncbi.nlm.nih.gov/compound/222284 |
|  | **HERB** | http://herb.ac.cn/Detail/?v=HBIN018278&label=Ingredient |
| CLR  1.32E+08 | **Pubchem** | https://pubchem.ncbi.nlm.nih.gov/compound/131698629 |
|  | **HERB** | http://herb.ac.cn/Detail/?v=HBIN021150&label=Ingredient |
| (E,E)-3,5-Di-O-caffeoylquinic acid  6474310 | **Pubchem** | https://pubchem.ncbi.nlm.nih.gov/compound/6474310 |
|  | **HERB** | http://herb.ac.cn/Detail/?v=HBIN024871&label=Ingredient |
| Isofucosterol  5281326 | **Pubchem** | https://pubchem.ncbi.nlm.nih.gov/compound/5281326 |
|  | **HERB** | http://herb.ac.cn/Detail/?v=HBIN030752&label=Ingredient |
| Neosurugatoxin  46173822 | **Pubchem** | https://pubchem.ncbi.nlm.nih.gov/compound/46173822 |
|  | **HERB** | http://herb.ac.cn/Detail/?v=HBIN036737&label=Ingredient |
| Campest-5-en-3beta-ol  173183 | **Pubchem** | https://pubchem.ncbi.nlm.nih.gov/compound/173183 |
|  | **HERB** | http://herb.ac.cn/Detail/?v=HBIN019471&label=Ingredient |
| Campesterol  173183 | **Pubchem** | https://pubchem.ncbi.nlm.nih.gov/compound/173183 |
|  | **HERB** | http://herb.ac.cn/Detail/?v=HBIN019475&label=Ingredient |
| Lysine  5962 | **Pubchem** | https://pubchem.ncbi.nlm.nih.gov/compound/5962 |
|  | **HERB** | http://herb.ac.cn/Detail/?v=HBIN034051&label=Ingredient |
| Avicularin  5490064 | **Pubchem** | https://pubchem.ncbi.nlm.nih.gov/compound/5490064 |
|  | **HERB** | http://herb.ac.cn/Detail/?v=HBIN017408&label=Ingredient |
| Matrine  91466 | **Pubchem** | https://pubchem.ncbi.nlm.nih.gov/compound/91466 |
|  | **HERB** | http://herb.ac.cn/Detail/?v=HBIN034558&label=Ingredient |
| Hyperoside  5281643 | **Pubchem** | https://pubchem.ncbi.nlm.nih.gov/compound/5281643 |
|  | **HERB** | http://herb.ac.cn/Detail/?v=HBIN029837&label=Ingredient |
| Japondipsaponin E1  11968646 | **Pubchem** | https://pubchem.ncbi.nlm.nih.gov/compound/11968646 |
|  | **HERB** | http://herb.ac.cn/Detail/?v=HBIN031428&label=Ingredient |
| Lupeol Acetate  92157 | **Pubchem** | https://pubchem.ncbi.nlm.nih.gov/compound/92157 |
|  | **HERB** | http://herb.ac.cn/Detail/?v=HBIN033753&label=Ingredient |
| Quercitrin  5280459 | **Pubchem** | https://pubchem.ncbi.nlm.nih.gov/compound/5280459 |
|  | **HERB** | http://herb.ac.cn/Detail/?v=HBIN041726&label=Ingredient |
| Histidine  6274 | **Pubchem** | https://pubchem.ncbi.nlm.nih.gov/compound/6274 |
|  | **HERB** | http://herb.ac.cn/Detail/?v=HBIN029439&label=Ingredient |
| Arginine  6322 | **Pubchem** | https://pubchem.ncbi.nlm.nih.gov/compound/6322 |
|  | **HERB** | http://herb.ac.cn/Detail/?v=HBIN016720&label=Ingredient |
| Sesamin | **Pubchem** | https://pubchem.ncbi.nlm.nih.gov/compound/72307 |
|  | **HERB** | http://herb.ac.cn/Detail/?v=HBIN043798&label=Ingredient |

**
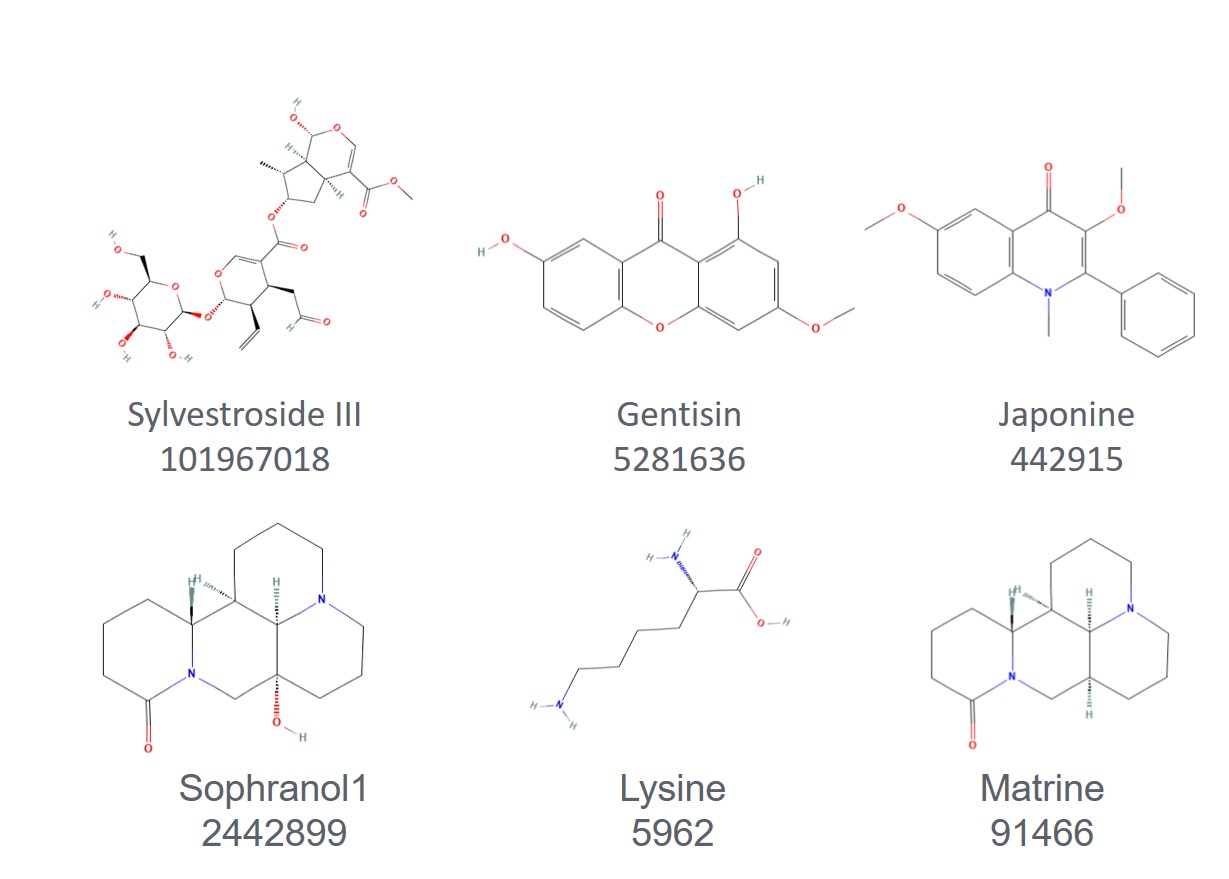
**

**FIGURE S3⏐** **Secondary structures of important compounds in KTH**


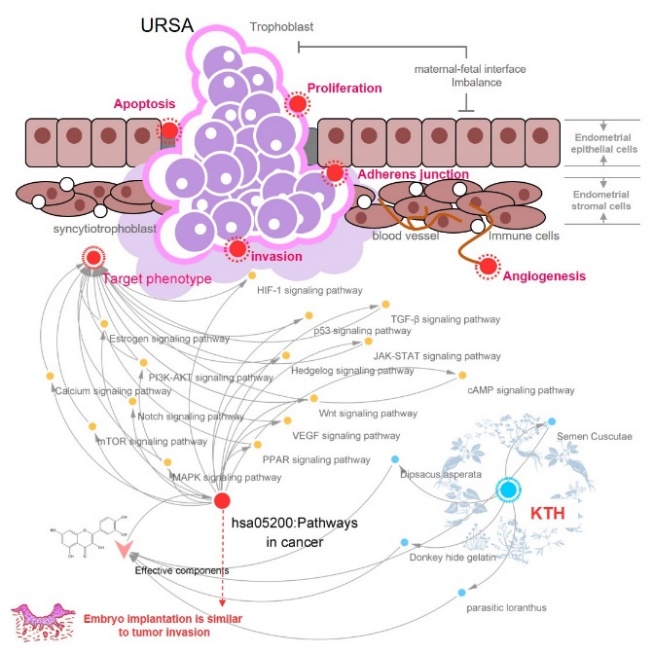


**FIGURE S4 ⏐**Summary diagram
