# Supplementary material for: A Potential Mechanism of Kidney-Tonifying Herbs Treating Unexplained Recurrent Spontaneous Abortion: Clinical Evidence From the Homogeneity of Embryo Implantation and Tumor Invasion
Source: Front Pharmacol. 2022 Jan 26;12:775245. doi: 10.3389/fphar.2021.775245 (PMC8826263; doi:10.3389/fphar.2021.775245)
Supplement: Supplementary file 2 [file DataSheet3.DOCX]

# SUPPLEMENTARY MATERIAL 3

## Quality of Evidence based on GRADE

We evaluated the certainty of the evidence of this study through GRADE pro. As a result, it was found that the certainty of evidence regarding KTH combined with medicine was low. The degradation of the certainty of the evidence was mainly due to the high risk of bias, the inconsistency among studies, and the imprecision of the findings.

**TABLE S****1⏐Quality of Evidence-Based on GRADE**

| **Outcome Indicators** | **No. of studies** | **No. of participants** | | **Effect**  **[95% CI]** | ***Heterogeneity***  ***I^2^*** | **Quality of**  **evidence** |
| --- | --- | --- | --- | --- | --- | --- |
|  |  | **Intervention**  **group** | **Control**  **group** |  |  |  |
| ***Clinical efficacy index***  **Clinical response rate** | | | | | | |
| TCM treatment VS conventional treatment | 3 | 200 | 184 | 1.04[0.66, 1.64] | 0% | 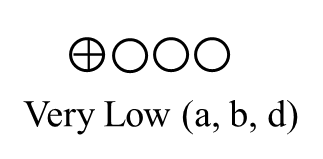 |
| Combined treatment VS conventional treatment | 7 | 384 | 377 | 4.36[2.91, 6.53] | 0% | 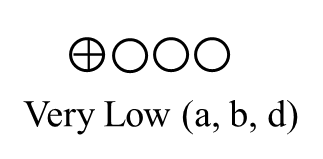 |
| **Total** | **8** | **584** | **561** | **2.39[1.79, 3.20]** | **63%** | 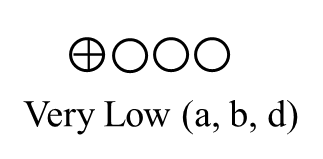 |
| **Pregnancy outcome** | **4** | **168** | **168** | **2.92[1.71,5.01]** | **0%** | **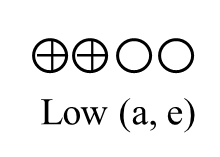** |
| ***Serum immunological parameters*** | | | | | | |
| **Interleukins-17(IL17)** | **2** | **89** | **80** | **-1.06[-1.38, -0.73]** | **0%** | **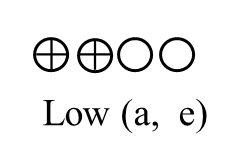*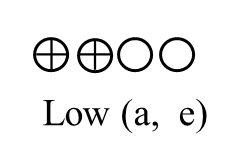*** |
| **Interleukins-4(IL4)** | **2** | **137** | **137** | **2.87[2.49, 3.25]** | **95%** | **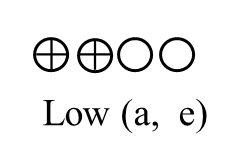** |
| **tumor necrosis factor alpha(TNFa)** | **2** | **132** | **132** | **-2.67[-3.01, -2.34]** | **0%** | **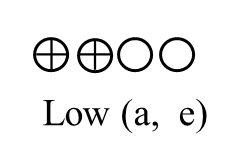** |
| ***Hormone*** ***levels*** | | | | | | |
| **Estradiol(E2)** | **4** | **227** | **227** | **2.12[0.27, 3.96]** | **98%** | **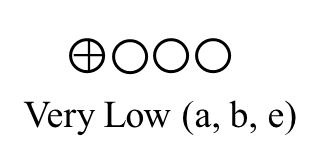** |
| **Progesterone(P)** | **5** | **174** | **171** | **1.41[0.72, 2.10]** | **87%** | **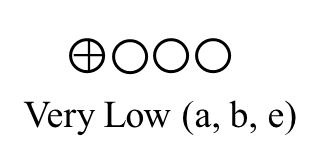** |
| **Human chorionic gonadotropin(HCG)** | **5** | **231** | **228** | **1.99[1.12, 2.86]** | **93%** | **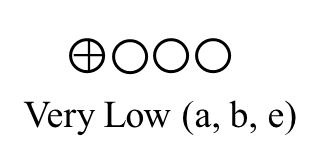** |

**Notes:** a: Download one level for serious risk of bias: failure to develop and apply appropriate eligibility criteria (inclusion of control population), flawed measurement of both exposure and outcome, failure to adequately control confounding, or Incomplete or inadequately short follow-up. b: Downgraded one level for serious inconsistent: inconsistency refers to an unexplained heterogeneity of results, which includes wide variance of point estimates across studies, minimal or no overlap of confidence intervals (CI), and statistical criteria, including tests of heterogeneity which test the null hypothesis that all studies have the same underlying magnitude of effect, have a low p-value (***P***<0.05), indicating to reject the null hypothesis. c: Downgraded one level for serious indirectness: including differences in the population (applicability), differences in interventions (applicability), differences in outcomes measures (surrogate outcomes), and indirect Comparisons. d: Downgraded one level for serious. e: Downgraded one level for serious imprecision: very small sample size.
