# Supplementary material for: A Potential Mechanism of Kidney-Tonifying Herbs Treating Unexplained Recurrent Spontaneous Abortion: Clinical Evidence From the Homogeneity of Embryo Implantation and Tumor Invasion
Source: Front Pharmacol. 2022 Jan 26;12:775245. doi: 10.3389/fphar.2021.775245 (PMC8826263; doi:10.3389/fphar.2021.775245)
Supplement: Supplementary file 3 [file DataSheet2.DOCX]

# SUPPLEMENTARY MATERIAL 2

## Supplement to bibliometric analysis methods


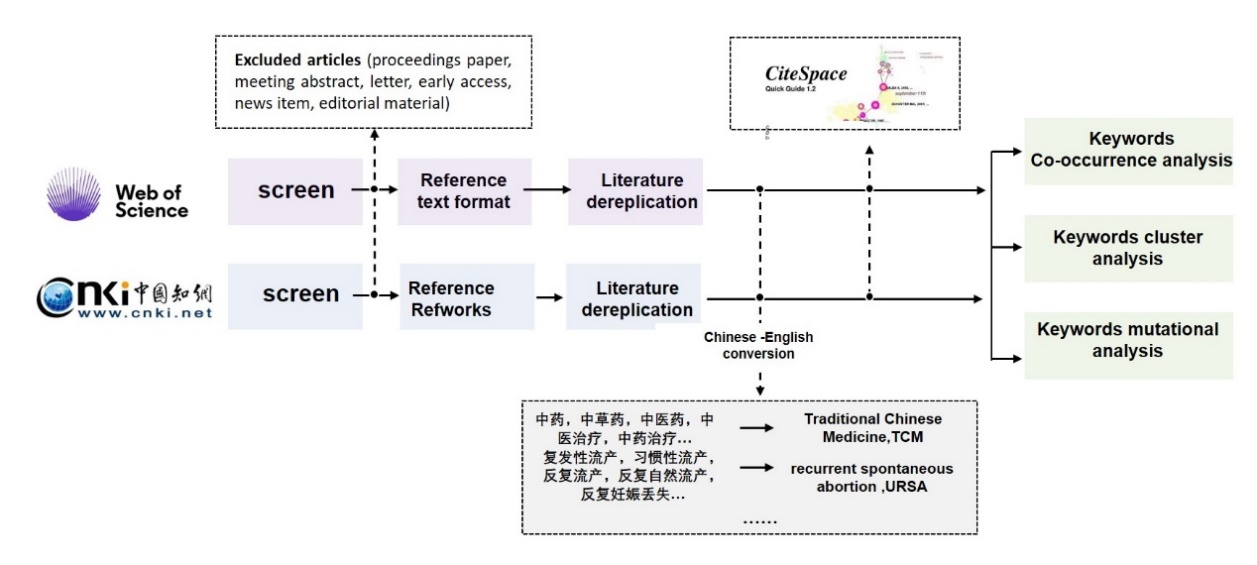
CiteSpace 5.8.R1 automatically eliminated duplicate literature records. The downloaded literature was evaluated by two reviewers to exclude literature unrelated to KTH and URSA; if two reviewers could not agree, a third reviewer was used.

**FIGURE S1⏐** **Flow chart of bibliometric analysis**

CiteSpace 5.8.R1, supplemented by Excel 2016 and GraphPad Prism 10.0 software, was used to display the trend in the number of articles published by year and the distribution of articles by a journal. Then CiteSpace V was used to perform a co-occurrence analysis and visualize the collaboration networks of the countries/institutes/authors/co-cited authors/ co-cited references /keywords. The time-slicing was set to Jan 1, 1991, to May 29, 2021, with1 one year per slice. G-index was selected as the criterion within Selection Criteria, and the k value was 25. Unfortunately, the Chinese literature records lack citation records, institutional records, citing records. We were only able to perform a keyword bibliometric analysis.
