# Supplementary material for: A Potential Mechanism of Kidney-Tonifying Herbs Treating Unexplained Recurrent Spontaneous Abortion: Clinical Evidence From the Homogeneity of Embryo Implantation and Tumor Invasion
Source: Front Pharmacol. 2022 Jan 26;12:775245. doi: 10.3389/fphar.2021.775245 (PMC8826263; doi:10.3389/fphar.2021.775245)
Supplement: Supplementary file 5 [file DataSheet1.DOCX]

# SUPPLEMENTARY MATERIAL 1

##
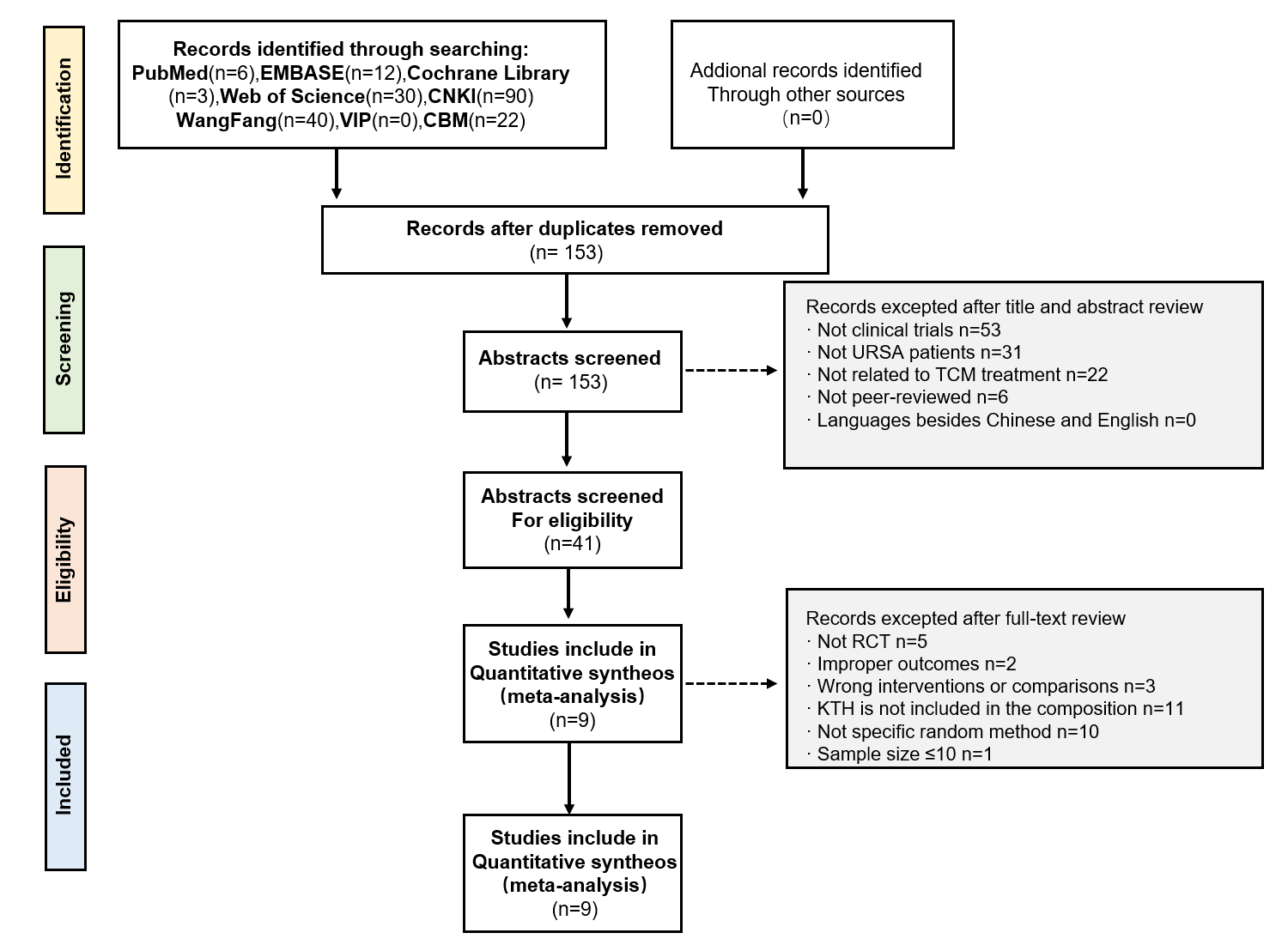
Flow chart of the literature screening and study selection

**FIGURE S1 ⏐**Flow chart of the literature screening and study selection.

## Literature search strategy based on PICOS principles

**TABLE S1⏐** Literature search strategy

| **PubMed search strategy( June, 2021)=6**  URL link :<https://pubmed.ncbi.nlm.nih.gov/>  **Expert Search :**  **#1 ((((((((("Abortion, Habitual"[Mesh]) OR (Habitual Abortion[Title/Abstract])) OR (Habitual Abortions[Title/Abstract])) OR (Miscarriage, Recurrent[Title/Abstract])) OR (Recurrent Miscarriage[Title/Abstract])) OR (Recurrent Miscarriages[Title/Abstract])) OR (Abortion, Recurrent[Title/Abstract])) OR (Recurrent Abortion[Title/Abstract])) OR (Recurrent Abortions[Title/Abstract])) OR (Recurrent Early Pregnancy Loss[Title/Abstract])**  **#2 (((((((( "Medicine, Chinese Traditional"[Mesh]) OR (Traditional Chinese Medicine[Title/Abstract])) OR (Chung I Hsueh[Title/Abstract])) OR (Hsueh, Chung I[Title/Abstract])) OR (Traditional Medicine, Chinese[Title/Abstract])) OR (Zhong Yi Xue[Title/Abstract])) OR (Chinese Traditional Medicine[Title/Abstract])) OR (Chinese Medicine, Traditional[Title/Abstract])) OR (((((((Tonifying kidney[Title/Abstract]) OR (Tonifying the kidney[Title/Abstract])) OR (Reinforce the kidney[Title/Abstract])) OR (Strengthening kidney[Title/Abstract])) OR (Nourishing kidney[Title/Abstract])) OR (Miscarriage prevention[Title/Abstract])) OR (Tocolysis[Title/Abstract]))**  **#3 (((("Pregnancy Rate"[Mesh]) OR (Pregnancy Outcomes[Title/Abstract])) OR (Outcome, Pregnancy[Title/Abstract])) OR (Outcomes, Pregnancy[Title/Abstract])) OR (Abortion outcome[Title/Abstract])**  **#4**  **((((((((((((((("Treatment Outcome"[Mesh]) OR (Outcome, Treatment[Title/Abstract])) OR (Patient-Relevant Outcome[Title/Abstract])) OR (Outcome, Patient-Relevant[Title/Abstract])) OR (Outcomes, Patient-Relevant[Title/Abstract])) OR (Patient Relevant Outcome[Title/Abstract])) OR (Patient-Relevant Outcomes[Title/Abstract])) OR (Clinical Effectiveness[Title/Abstract])) OR (Effectiveness, Clinical[Title/Abstract])) OR (Treatment Effectiveness[Title/Abstract])) OR (Effectiveness, Treatment[Title/Abstract])) OR (Rehabilitation Outcome[Title/Abstract])) OR (Outcome, Rehabilitation[Title/Abstract])) OR (Treatment Efficacy[Title/Abstract])) OR (Efficacy, Treatment[Title/Abstract])) OR (Clinical Efficacy[Title/Abstract])**  **#5 ((((((Tonifying kidney[Title/Abstract]) OR (Tonifying the kidney[Title/Abstract])) OR (Reinforce the kidney[Title/Abstract])) OR (Strengthening kidney[Title/Abstract])) OR (Nourishing kidney[Title/Abstract])) OR (Miscarriage prevention[Title/Abstract])) OR (Tocolysis[Title/Abstract])**  **#6 ((((((("Medicine, Chinese Traditional"[Mesh]) OR (Traditional Chinese Medicine[Title/Abstract])) OR (Chung I Hsueh[Title/Abstract])) OR (Hsueh, Chung I[Title/Abstract])) OR (Traditional Medicine, Chinese[Title/Abstract])) OR (Zhong Yi Xue[Title/Abstract])) OR (Chinese Traditional Medicine[Title/Abstract])) OR (Chinese Medicine, Traditional[Title/Abstract])**  **#7 #3 OR #4 OR #5 OR #6**  **#8 (("Randomized Controlled Trial" [Publication Type]) OR "Controlled Clinical Trial" [Publication Type]) OR "Equivalence Trial" [Publication Type]**  **#9 #1 AND #2 AND #7 AND #8** |
| --- |
| **Cocharne search strategy(June, 2021)=3**  URL link :<https://www.cochranelibrary.com/>  **Expert Search :**  #1 MeSH descriptor: [Abortion, Habitual] explode all trees  #2 (Habitual Abortion):ti,ab,kw OR (Habitual Abortions):ti,ab,kw OR (Miscarriage, Recurren):ti,ab,kw OR (Recurrent Miscarriage):ti,ab,kw OR (Abortion, Recurrent):ti,ab,kw OR (Recurrent Miscarriage):ti,ab,kw OR (Recurrent Abortion):ti,ab,kw OR (Recurrent Abortions):ti,ab,kw AND (Recurrent Early Pregnancy Loss):ti,ab,kw (Word variations have been searched)  #3 MeSH descriptor: [Abortion, Spontaneous] explode all trees  #4 (Miscarriage):ti,ab,kw OR (Miscarriages):ti,ab,kw OR (Spontaneous Abortion):ti,ab,kw OR (Spontaneous Abortions):ti,ab,kw OR (Abortions, Spontaneous):ti,ab,kw OR (Pregnancy Losses, Early):ti,ab,kw ( Early Pregnancy Loss):ti,ab,kw OR ( Losses, Early Pregnancy):ti,ab,kw OR (Loss, Early Pregnancy):ti,ab,kw OR (Early Pregnancy Losses):ti,ab,kw OR (Loss, Early Pregnancy):ti,ab,kw OR (Pregnancy Loss, Early):ti,ab,kw (Word variations have been searched)  #5 #1 OR #2 OR #3 OR #4 OR #4  #6 MeSH descriptor: [kidney] explode all trees  #7 MeSH descriptor: [Tocolysis] explode all trees  #8 MeSH descriptor: [Medicine, Chinese Traditional] explode all trees  #9 (Traditional Chinese Medicine):ti,ab,kw OR (Chung I Hsueh  ):ti,ab,kw OR (Hsueh, Chung I):ti,ab,kw OR (Traditional Medicine, Chinese):ti,ab,kw OR (Zhong Yi Xue):ti,ab,kw OR (Chinese Traditional Medicine):ti,ab,kw OR (Chinese Medicine, Traditional):ti,ab,kw (Word variations have been searched)  #10 #6 OR #7 OR #8 OR #9  #11 MeSH descriptor: [Treatment Outcome] explode all trees  #12 MeSH descriptor: [Pregnancy Rate] explode all trees  #13 (Patient Relevant Outcome):ti,ab,kw OR (Patient-relevant Outcome):ti,ab,kw OR (Outcome, Patient-Relevant):ti,ab,kw OR (Patient-Relevant Outcomes):ti,ab,kw OR (Outcomes, Patient-Relevant):ti,ab,kw OR (Effectiveness, Clinical):ti,ab,kw OR (Clinical Effectiveness):ti,ab,kw OR (Effectiveness, Treatment):ti,ab,kw OR (Treatment Effectiveness):ti,ab,kw OR (Efficacy, Clinical):ti,ab,kw OR (Clinical Efficacy):ti,ab,kw OR (Treatment Efficacy):ti,ab,kw OR (Efficacy, Treatment):ti,ab,kw OR (Outcome Treatment):ti,ab,kw OR (Outcome, Rehabilitation):ti,ab,kw OR (Rehabilitation Outcome):ti,ab,kw (Word variations have been searched)  #14 (Pregnancy Rates, Live-Birth):ti,ab,kw OR (Live-Birth):ti,ab,kw OR (Life-Birth Pregnancy Rate):ti,ab,kw OR (Pregnancy Rate, Live-Birth):ti,ab,kw OR (Live-Birth Pregnancy Rates):ti,ab,kw OR (Live Birth Pregnancy Rate):ti,ab,kw OR (Pregnancy Rate, Live Birth):ti,ab,kw OR (Rates, Live-Birth Pregnancy):ti,ab,kw OR (Rate, Live-Birth Pregnancy):ti,ab,kw OR (Rate, Pregnancy):ti,ab,kw OR (Pregnancy Rates):ti,ab,kw OR (Rates, Pregnancy):ti,ab,kw (Word variations have been searched)  #15 #11 OR #12 OR #13 OR #14  #16 MeSH descriptor: [Randomized Controlled Trial] explode all trees  #17 #5 AND #10 AND #15 AND #16 |
| **Web of Science** **search strategy(June, 2021)=30**  URL link: <https://www.webofscience.com/wos/woscc/basic-search>  **Expert Search :**  **(((((((((((TS=(Abortion, Habitual)) OR TS=(Habitual Abortion)) AND TS=(Habitual Abortions))) OR TS=(Miscarriage, Recurrent)) OR TS=(Recurrent Miscarriage))) OR TS=(Recurrent Miscarriages)) OR TS=(Abortion, Recurrent)) OR TS=(Recurrent Abortion)) OR TS=(Recurrent Abortions)) OR TS=(Recurrent Early Pregnancy Loss) AND (((((((TS=(Medicine, Chinese Traditional)) OR TS=(Traditional Chinese Medicine)) OR TS=(Chung I Hsueh)) OR TS=(Hsueh, Chung I)) OR TS=(Traditional Medicine, Chinese)) OR TS=(Zhong Yi Xue)) OR TS=(Chinese Traditional Medicine)) OR TS=(Chinese Medicine, Traditional) AND (((((((TS=(Tonifying kidney )) OR TS=(Tonifying the kidney)) OR TS=(Reinforce the kidney)) OR TS=(Strengthening kidney )) OR TS=(Nourishing kidney))) OR TS=(Miscarriage prevention)) OR TS=(Tocolysis) AND ((((((((((((((((((((((((((((((((TS=(Treatment Outcome)) OR TS=(Outcome, Treatment))) OR TS=(Patient-Relevant Outcome)) OR TS=(Outcome, Patient-Relevant)) OR TS=(Outcomes, Patient-Relevant)) OR TS=(Patient Relevant Outcome)) OR TS=(Patient-Relevant Outcomes)) OR TS=(Clinical Effectiveness)) OR TS=(Effectiveness, Clinical)) OR TS=(Treatment Effectiveness)) OR TS=(Effectiveness, Treatment)) OR TS=(Rehabilitation Outcome)) OR TS=(Outcome, Rehabilitation)) OR TS=(Treatment Efficacy)) OR TS=(Efficacy, Treatment)) OR TS=(Clinical Efficacy)) OR TS=(Pregnancy Rate)) OR TS=(Rates, Pregnancy)) OR TS=(Pregnancy Rates)) OR TS=(Rate, Pregnancy)) OR TS=(Pregnancy Rate, Live-Birth)) OR TS=(Live-Birth Pregnancy Rates)) OR TS=(Pregnancy Rate, Live Birth)) OR TS=(Pregnancy Rates, Live-Birth)) OR TS=(Rate, Live-Birth Pregnancy)) OR TS=(Rates, Live-Birth Pregnancy)) OR TS=(Live-Birth Pregnancy Rate)) OR TS=(Live Birth Pregnancy Rate)) OR TS=(Pregnancy Outcomes)) OR TS=(Outcome, Pregnancy)) OR TS=(Outcomes, Pregnancy)) OR TS=(Abortion outcome) AND ((TS=(Randomized Controlled Trial)) OR TS=(Controlled Clinical Trial)) OR TS=(Equivalence Trial)** |
| **EMBASE search strategy search strategy( June, 2021)=12**  URL link :<https://www.embase.com/>  **Expert Search :**  ('recurrent abortion'/exp OR 'Habitual Abortion':ti,ab,kw OR ' Miscarriage, Recurrent ':ti,ab,kw OR 'Recurrent Miscarriage ':ti,ab,kw OR ' Abortion, Recurrent ':ti,ab,kw OR ' Recurrent Miscarriage ':ti,ab,kw OR ' Recurrent Abortion ':ti,ab,kw OR ' Recurrent Abortions ':ti,ab,kw OR ' Recurrent Early Pregnancy Loss ':ti,ab OR ' Abortion, Spontaneous '/exp OR ' Miscarriage ':ti,ab OR ' Spontaneous Abortion ':ti,ab,kw OR ' Spontaneous Abortions ':ti,ab,kw OR ' Abortions, Spontaneous ':ti,ab,kw OR ' Pregnancy Losses, Early ':ti,ab,kw OR ' Early Pregnancy Loss ':ti,ab,kw OR ' Losses, Early Pregnancy ':ti,ab,kw OR ' Loss, Early Pregnancy ':ti,ab OR ' Early Pregnancy Losses ':ti,ab OR ' Loss, Early Pregnancy ':ti,ab OR ' Pregnancy Loss, Early ':ti,ab) AND (' kidney '/exp OR ' Tocolysis '/exp OR ' Medicine, Chinese Traditional '/exp OR Traditional Chinese Medicine:ti,ab OR ' Chung I Hsueh ':ti,ab,kw OR ' Hsueh, Chung I ':ti,ab,kw OR ' Traditional Medicine, Chinese ':ti,ab OR ' Zhong Yi Xue ':ti,ab OR 'Chinese Traditional Medicine:ti,ab OR ' Losses, Early Pregnancy ':ti,ab,kw OR ' Loss, Early Pregnancy ':ti,ab OR ' Early Pregnancy Losses ':ti,ab OR ' Loss, Early Pregnancy ':ti,ab OR ' Pregnancy Loss, Early ':ti,ab) AND (' kidney '/exp OR ' Tocolysis '/exp OR ' Medicine, Chinese Traditional '/exp OR Traditional Chinese Medicine:ti,ab AND (' Treatment Outcome '/exp OR ' Pregnancy Rate '/exp OR ' Patient Relevant Outcome ':ti,ab OR ' uterine fundus ':ti,ab OR ' Outcome, Patient-Relevant ':ti,ab OR ' Patient-Relevant Outcomes ':ti,ab OR ' Outcomes, Patient-Relevant ':ti,ab OR ' Effectiveness, Clinical ':ti,ab OR ' Clinical Effectiveness ':ti,ab OR' Treatment Effectiveness ':ti,ab,kw OR ' Efficacy, Clinical ':ti,ab OR ' Clinical Efficacy ':ti,ab OR ' Treatment Efficacy ':ti,ab OR ' Efficacy, Treatment ':ti,ab) OR ' Outcome, Rehabilitation ':ti,ab OR' Rehabilitation Outcome ':ti,ab,kw OR' Pregnancy Rates, Live-Birth ':ti,ab OR ' Live-Birth ':ti,ab OR' Life-Birth Pregnancy Rate ':ti,ab,kw OR ' Pregnancy Rate, Live-Birth ':ti,ab OR ' Live-Birth Pregnancy Rates ':ti,ab OR ' Live Birth Pregnancy Rate ':ti,ab OR ' Pregnancy Rate, Live Birth ':ti,ab) OR ' O Rates, Live-Birth Pregnancy ':ti,ab OR' Rate, Live-Birth Pregnancy ':ti,ab,kw OR ' Rate, Pregnancy ':ti,ab OR ' Pregnancy Rates ':ti,ab) OR ' Rates, Pregnancy ':ti,ab AND (' Randomized Controlled Trial)'/exp OR ' Controlled Clinical Trial ' OR ' Equivalence Trial:ti,ab) |
| **CNKI search strategy(June 8, 2021) =90**  URL link :<https://kns.cnki.net/>  Su% = theme, TKA = article summary  **Expert Search :**  (SU=不明原因+原因不明) AND (SU=复发性流产+反复流产+反复早期流产+反复妊娠丢失+习惯性流产+多次流产) AND (SU=补肾+固肾+滋肾+中医+中药+中医药+安胎) AND (TKA=临床疗效+妊娠率+妊娠结局+临床反应率+安胎结局) AND(TKA=随机对照试验+临床研究+临床试验+RCT) |
| **Wanfang Database search strategy(June 8, 2021) =40**  URL link :<https://s.wanfangdata.com.cn/>  **Expert Search :**  ((不明原因 OR 原因不明) AND (复发性流产 OR 反复流产 OR 反复早期流产 OR 反复妊娠丢失 OR 习惯性流产 OR 多次流产) AND (补肾 OR 固肾 OR 滋肾 OR 中医 OR 中药 OR 中医药 OR 安胎) AND (临床疗效 OR 妊娠率 OR 妊娠结局 OR 临床反应率 OR 安胎结局) AND (随机对照试验 OR 临床研究 OR 临床试验 OR RCT)) |
| **CBM search strategy((June 8, 2021) =22**  URL link :<http://www.sinomed.ac.cn/>  **Expert Search :**  ("不明原因"[常用字段:智能] OR "原因不明"[常用字段:智能]) AND ("复发性流产"[常用字段:智能] OR "反复流产"[常用字段:智能] OR "反复早期流产"[常用字段:智能] OR "反复妊娠丢失"[常用字段:智能] OR "习惯性流产"[常用字段:智能] OR "多次流产"[常用字段:智能]) AND( "补肾"[常用字段:智能] OR "固肾"[常用字段:智能] OR "滋肾"[常用字段:智能] OR "中医"[常用字段:智能] OR "中医药"[常用字段:智能] OR "安胎"[常用字段:智能]) AND( "临床疗效"[常用字段:智能] OR "妊娠率"[常用字段:智能] OR "妊娠结局"[常用字段:智能] OR "子宫内膜容受"[常用字段:智能] OR "子宫动脉"[常用字段:智能] OR "临床反应率"[常用字段:智能] OR "安胎结局"[常用字段:智能]) AND( "随机对照试验"[常用字段:智能] OR "临床研究"[常用字段:智能] OR "临床试验"[常用字段:智能] OR "RCT"[常用字段:智能]) |
| **VIP search strategy(June 8, 2021) =0**  URL link : <http://qikan.cqvip.com/>  **Expert Search :**  (题名或关键词:(不明原因 OR 原因不明)) AND (题名或关键词:(复发性流产 OR 反复流产 OR 反复早期流产 OR 反复妊娠丢失 OR 习惯性流产 OR 多次流产)) AND (题名或关键词:(补肾 OR 固肾 OR 滋肾 OR 中医 OR 中药 OR 中医药 OR 安胎)) AND (题名或关键词:(临床疗效 OR 妊娠率 OR 妊娠结局 OR 临床反应率 OR 安胎结局)) AND (题名或关键词: (随机对照试验 OR 临床研究 OR 临床试验 OR RCT)) |
